# Body mass index and waist circumference in relation to the risk of 26 types of cancer: a prospective cohort study of 3.5 million adults in Spain

**DOI:** 10.1186/s12916-020-01877-3

**Published:** 2021-01-14

**Authors:** Martina Recalde, Veronica Davila-Batista, Yesika Díaz, Michael Leitzmann, Isabelle Romieu, Heinz Freisling, Talita Duarte-Salles

**Affiliations:** 1 Fundació Institut Universitari per a la recerca a l’Atenció Primària de Salut Jordi Gol i Gurina (IDIAPJGol), Gran Via Corts Catalanes, 587 àtic, 08007 Barcelona, Spain; 2 Universitat Autònoma de Barcelona, Campus de Bellaterra, 08193 Bellaterra, Spain; 3 International Agency for Research on Cancer (IARC-WHO), 150 Cours Albert Thomas, 69008 Lyon, France; 4 Consortium for Biomedical Research in Epidemiology and Public Health (CIBERESP), 28029 Madrid, Spain; 5 Department of Epidemiology and Preventive Medicine, University of Regensburg, Franz-Josef-Strauss-Allee 11, 93053 Regensburg, Germany; 6 Center for Research on Population Health, National Institute of Public Health, Mexico City, Mexico; 7 Hubert Department of Global Health, Emory University, Atlanta, GA USA

**Keywords:** Body mass index, Waist circumference, Body size, Body fat distribution, Adiposity, Obesity, Cancer, Electronic health records

## Abstract

**Background:**

A high body mass index (BMI) has been associated with increased risk of several cancers; however, whether BMI is related to a larger number of cancers than currently recognized is unclear. Moreover, whether waist circumference (WC) is more strongly associated with specific cancers than BMI is not well established. We aimed to investigate the associations between BMI and 26 cancers accounting for non-linearity and residual confounding by smoking status as well as to compare cancer risk estimates between BMI and WC.

**Methods:**

Prospective cohort study with population-based electronic health records from Catalonia, Spain. We included 3,658,417 adults aged ≥ 18 years and free of cancer at baseline between 2006 and 2017. Our main outcome measures were cause-specific hazard ratios (HRs) with 99% confidence intervals (CIs) for incident cancer at 26 anatomical sites.

**Results:**

After a median follow-up time of 8.3 years, 202,837 participants were diagnosed with cancer. A higher BMI was positively associated with risk of nine cancers (corpus uteri, kidney, gallbladder, thyroid, colorectal, breast post-menopausal, multiple myeloma, leukemia, non-Hodgkin lymphoma) and was positively associated with three additional cancers among never smokers (head and neck, brain and central nervous system, Hodgkin lymphoma). The respective HRs (per 5 kg/m^2^ increment) ranged from 1.04 (99%CI 1.01 to 1.08) for non-Hodgkin lymphoma to 1.49 (1.45 to 1.53) for corpus uteri cancer. While BMI was negatively associated to five cancer types in the linear analyses of the overall population, accounting for non-linearity revealed that BMI was associated to prostate cancer in a U-shaped manner and to head and neck, esophagus, larynx, and trachea, bronchus and lung cancers in an L-shaped fashion, suggesting that low BMIs are an approximation of heavy smoking. Of the 291,305 participants with a WC measurement, 27,837 were diagnosed with cancer. The 99%CIs of the BMI and WC point estimates (per 1 standard deviation increment) overlapped for all cancers.

**Conclusions:**

In this large Southern European study, a higher BMI was associated with increased risk of twelve cancers, including four hematological and head and neck (only among never smokers) cancers. Furthermore, BMI and WC showed comparable estimates of cancer risk associated with adiposity.

**Supplementary information:**

**Supplementary information** accompanies this paper at 10.1186/s12916-020-01877-3.

## Background

The prevalence of obesity worldwide has nearly tripled over the past three decades, reaching 650 million adults in 2016 [1]. Body mass index (BMI), the most common indicator of general adiposity, has been convincingly associated with at least 12 cancer types [2]. Results from previous large cohort studies suggest that BMI is associated with a larger number of cancer types than currently recognized and that some of those associations may be non-linear [3, 4]. However, the main limitations of available studies include limited adjustment for potential confounding, reliance on self-reported weight and height, and lack of generalizability to different populations. Furthermore, although conducting analyses stratified by smoking status is critical to provide unbiased estimates of the impact of obesity on cancer risk [4, 5], many studies failed to present results stratified by smoking status, in part due to insufficient statistical power [3].

In addition, whether BMI as a sole indicator of general adiposity fully captures the complex association between adiposity and cancer risk is still in dispute. Central adiposity, typically assessed using waist circumference (WC), has been suggested to increase the risk of several cancer types and to better discriminate risk associated with obesity for colon and breast post-menopausal cancers [6–8]. However, only few studies have systematically compared the effect estimates of BMI and WC for multiple site-specific cancers, and none have studied less frequently occurring cancer types [9, 10].

The primary objective of the current study was to investigate associations between BMI and the risk of 26 types of cancer accounting for non-linearity and residual confounding by smoking status. Our secondary objective was to compare risk estimates for general (BMI) and central (WC) adiposity in relation to the risk of 26 cancer types.

## Methods

### Study design, setting, and data sources

We performed a cohort study with prospectively collected data from the Information System for Research in Primary Care (SIDIAP; www.sidiap.org), from January 1, 2006, until December 31, 2018. SIDIAP includes routinely recorded information by health professionals from 287 primary care centers in Catalonia, a region in Northeastern Spain [11, 12]. SIDIAP contains anonymized records for approximately six million people (80% of the Catalan population) and is representative of the Catalan population in terms of age, sex, and geographic distribution [12]. It includes high-quality data on anthropometric measurements, disease diagnoses (International Classification for Diseases, 10th revision [ICD-10]), prescription and dispensation of drugs, laboratory tests, and demographic and lifestyle information. Further, SIDIAP is linked to the Minimum Basic Dataset (CMBD in Spanish), a population-based registry that includes hospital discharge information in Spain [13].

### Participants

**Fig. 1:**
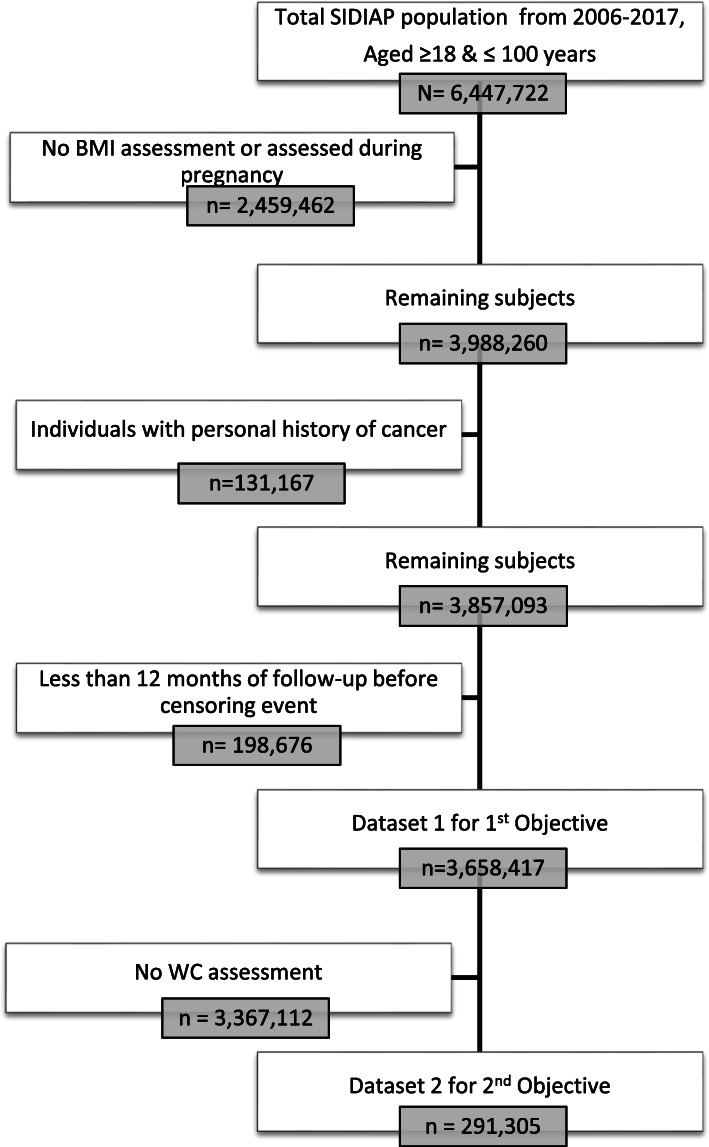
Flow chart showing the creation of the study’s datasets with the participants’ inclusion and exclusion criteria. Notes: 221 individuals were aged above 100 years at the time of their first available BMI measurement. Abbreviations: BMI, body mass index; SIDIAP, Information System for Research in Primary Care; WC, waist circumference

For the primary objective, we included all participants aged ≥ 18 years with a valid BMI (weight (kg)/height (m)^2^ between 15 and 60 kg/m^2^) recorded between January 1, 2006, and December 31, 2017, and subsequent eligible follow-up time (minimum of 1 year). The study’s index date was the date of the first BMI assessment during this period. We followed participants from the study index date until first incident (primary) cancer diagnosis, death, transferal out of the SIDIAP, or until the end of the study period (December 31, 2018). We excluded individuals who were older than 100 years of age at index date, had a BMI assessment only available during pregnancy (from the 3rd month of pregnancy until 2 months after delivery), had any record of cancer before the study index date, or complied with any of the end-of-follow-up criteria described above before attaining 12 months of follow-up to avoid reverse causality (Fig. 1, dataset 1). For our secondary objective, we included an additional eligibility criterion, which was to have a valid WC assessment (WC values ≥ 40 cm and ≤ 160 cm) no more than 5 years previous to or 1 year later than the index date (first BMI measurement recorded) (Fig. 1, dataset 2). If a participant had more than one WC measurement available, we selected the closest one to the index date. Figure 1 shows the flow chart of inclusion and exclusion criteria for each study objective.

### Assessment of anthropometric indicators and covariates

For our primary objective, the exposure of interest was BMI as a continuous variable (in kg/m^2^). BMI was automatically calculated through a computer program (“Estació clínica d’atenció primària”) after general practitioners (GPs) or nurses entered the weight (kg) and height (cm) of patients they directly assessed in a standardized manner [14]. For participants without information from that computer program, we calculated the BMI using weight and height data available in their health records (if height was not available on the same date as the weight measurement, we calculated the individuals’ mean height using all available measurements in their health records during adulthood (≥ 18 years) and we chose the closest real height value to the mean). For our secondary objective, we additionally considered WC as an exposure; this indicator was routinely measured by trained health professionals (GPs and nurses) who follow a measurement protocol [15]. WC was measured at the umbilical level, midway between the anterior superior iliac spine and the inferior border of the rib while participants were standing.

We also extracted information on sex (women, men), age (in years), and geographic region of nationality (Spain, European [non-Spanish], Africa, America, and Asia). We assessed socioeconomic status in urban areas using the “Mortalidad en áreas pequeñas Españolas y Desigualdades Socioeconómicas y Ambientales” (MEDEA) deprivation index, which is calculated at the census tract level and was categorized into quintiles by the SIDIAP for anonymization purposes [16]. The first and the fifth quintiles represent the least and most deprived groups of the population living in urban areas of Catalonia, respectively. We included a rural category since the MEDEA index was not available for participants living in those areas. We also extracted information on smoking status (never, former, or current smoker) and alcohol intake (none, low or high). If a participant had more than one record of smoking status and alcohol intake available, we selected the one closest to the index date within a 6-year period (5 years before and 1 year after the first BMI measurement). For type 2 diabetes, we considered any registry of a GP diagnosis (ICD-10 code E11) before the index date. For women, we included information on menopausal status and hormonal replacement therapy (HRT) use, the definitions of which can be consulted in Additional file 1: Appendix S1.

### Ascertainment of cancer cases

We considered first incident cancer diagnoses as the outcomes of interest. We identified outcomes using ICD-10 codes in the SIDIAP database and ICD-9 codes in the CMBD from January 1, 2007, to December 31, 2018. We mapped ICD-9 diagnosis codes to ICD-10 using available conversion codes (eCIEMaps v3.1.9) which are provided in Additional file 1: Table S1. We used the following cancer types as outcomes: head and neck; esophagus; stomach; colorectal; liver; gallbladder and biliary tract; pancreas; larynx; trachea, bronchus, and lung; bone and articular cartilage; malignant melanoma of skin; breast (which we categorized into pre- and post-menopausal due to well-established evidence indicating different BMI relations) [17]; cervix uteri; corpus uteri; ovary; prostate; testis; kidney; bladder; brain and central nervous system (CNS); thyroid; Hodgkin lymphoma; non-Hodgkin lymphoma; multiple myeloma; and leukemia. All cancer diagnoses in the SIDIAP including the CMBD have been previously validated [18].

### Statistical analysis

We described the number of excluded individuals in each step of the creation of the main dataset. We presented the overall baseline characteristics of the study participants and by the World Health Organization (WHO) BMI categories: underweight or normal weight (BMI < 18.5 kg/m^2^ and between ≥ 18.5 and < 25 kg/m^2^), overweight (BMI ≥ 25 and < 30 kg/m^2^), and obesity (BMI ≥ 30 kg/m^2^).

We fitted Cox proportional hazard models with age as the time metric to estimate cause-specific hazard ratios (HR) and 99% confidence intervals (CI) for the relation between BMI and risk of each cancer type. We stratified all models by age (5-year categories) and sex to reduce the sensitivity to violations of the proportional hazards assumption. The first (basic) model included BMI only (model 1) and the second (multivariable-adjusted) model further adjusted for smoking status, alcohol intake, type 2 diabetes, socioeconomic status, and nationality (model 2). A directed acyclic graph was used to guide decisions on the control for confounding (Additional file 1: Fig. S1) [19]. We used a missing category for variables with missing data.

Firstly, we investigated potential non-linear associations between BMI and risk of each cancer. We considered non-linearity in BMI by fitting models using restricted cubic splines for BMI with 3 knots (placed at the 10th, 50th, and 90th percentiles) or 5 knots (placed at the 5th, 27.5th, 50th, 72.5th, and 95th percentiles). We evaluated linearity by comparing the Akaike information criterion of models with restricted splines to the model with BMI as a linear term in combination with a Wald test linearity hypothesis [20, 21]. To assess residual confounding by smoking, we re-run the multivariable-adjusted (adjusted for alcohol intake, type 2 diabetes, socioeconomic status, and nationality) models among never smokers for cancers for which we found evidence of non-linearity.

Secondly, we fitted model 2 with BMI as a linear term to estimate HRs of the relation between BMI (per 5 kg/m^2^ increment) and risk of each cancer type. Again, we re-run the multivariable-adjusted models (adjusted for alcohol intake, type 2 diabetes, socioeconomic status, and nationality) only among participants who reported having never smoked to explore residual confounding by smoking.

In the subsample of participants who had information on both BMI and WC (Fig. 1, dataset 2), we compared risk estimates for general (BMI) and central (WC) adiposity in relation to the risk of 26 cancers by fitting Cox proportional hazard models (one for each adiposity indicator) with age as the time metric. We estimated HRs and 99% CIs per 1 standard deviation (SD) increment of adiposity indicators (BMI and WC) to allow comparability between both estimates [9]. We considered estimates different if the 99% CIs of the point estimates of each adiposity indicator did not overlap. We adjusted the statistical models for the same variables as in model 2, and we used the same end of follow-up definition. We only analyzed cancer types for which we ascertained at least 100 cancer cases.

### Model-checking and sensitivity analyses

For all models, we checked the proportional hazard assumption by using the Schoenfeld test of proportionality and by visual inspection of the scaled Schoenfeld residuals [22].

We assessed the robustness of our primary objective findings by performing six sensitivity analyses. First, we accounted for residual selection bias by additionally adjusting model 2 for the number of GP consultations in the year of the index date because participants who see their GP more often may have different health behaviors than those who see their GP less often. Second, we explored potential outcome misclassification by restricting the analyses to specific regions of Catalonia where we had access to population-based or hospital cancer registries. We considered as cancer cases only those who had the same diagnosis in the SIDIAP and a cancer registry. Third, we addressed potential reverse causality (i.e., undiagnosed cancer affecting BMI) by extending the minimum follow-up time (of 1 year in the main analyses) to 2 and 4 years. Fourth, we strengthened the validity of our results by performing multiple imputations (using the fully conditional specification approach, with 10 imputed data sets created) to deal with missing values of model 2 covariates [23, 24]. Fifth, we avoided confounding in the analyses of BMI and specific cancer types by re-running model 2, additionally adjusting for HRT use in post-menopausal women [women-only cancers] and excluding participants with a diagnosis of chronic hepatitis B/C [liver cancer risk factor] or a helicobacter pylori infection [stomach cancer risk factor]). Finally, to investigate to which extent the relationships between BMI and risk of each cancer type represents an effect of weight, height, or both weight and height, we re-ran the multivariable-adjusted models (model 2) with height and weight as the main exposures, mutually adjusted for each other.

To assess the robustness of our secondary findings, we performed two sensitivity analyses. We re-ran the analyses that compared BMI and WC in relation to cancer risk with mutual adjustment for both adiposity indicators using residuals of WC and BMI (e.g., we regressed WC on BMI, and we included the residuals from this analysis in the model using BMI as an indicator of general adiposity) to assess if this added valuable information to fully capture adiposity [9]. Finally, we added height as an adjustment variable to the analyses that compared BMI and WC in relation to cancer risk.

The a priori level of statistical significance was set at a 2-sided *P* value of 0.01 for all analyses. We used STATA version 15.1 (College Station, TX, USA) for data analysis and R version 3.5.0 for data visualization.

We obtained approval from the Clinical Research Ethics Committee of the IDIAPJGol (project code: P14/074) to perform this study. 

## Results

Of the 6,447,722 individuals aged between ≥ 18 and ≤ 100 years in the SIDIAP population, 2,459,462 were excluded due to the unavailability of a valid BMI, 131,167 due to personal history of cancer, and 198,676 due to less than 12 months of follow-up (Fig. 1). A total of 3,658,417 participants constituted the primary dataset of this study for whom follow-up ended at a median of 8.3 years (interquartile range [IQR] 5–11) after study entry. In total, 202,828 [5.6%] individuals were diagnosed with cancer over the study period (Table 1). Among all participants, 55% were women, the median age at inclusion was 46 years (IQR 32–61), and the median BMI was 26.3 kg/m^2^ (IQR 23–30). When stratifying participants by WHO categories of BMI, the median follow-up and age increased with increasing categories of BMI. There were fewer participants from deprived areas and more current smokers in the underweight and normal weight category compared to those in the obesity category (Table 1). Compared to the overall SIDIAP adult population, the individuals included in this study were more likely to be women and older, as well as to have more comorbidities and complete information on lifestyle factors (the characteristics of the included and excluded individuals can be consulted in Additional file 1: Table S2).

**Table 1:** Baseline characteristics of the study participants included in the analyses of the first objective (dataset 1) by body mass index categories and of the second objective (dataset 2)

	Dataset 1, ***N*** (%)				Dataset 2, ***N*** (%)
	Under and normal weight (BMI < 25)	Overweight (BMI ≥ 25 and < 30)	Obese (BMI ≥ 30)	BMI total	WC total
**Characteristic**	1,436,991 (39.3)	1,326,642 (36.3)	894,784 (24.4)	3,658,417 (100)	291,305 (100)
**Follow-up (in years)**^**a,b**^	7.7 (4.4–10.6)	8.5 (5.0–11.3)	9.1 (5.6–11.8)	8.3 (4.9–11.2)	9.9 (6.9–11.9)
**Visits to health center**^**b,c**^	5 (3–9)	7 (4–12)	8 (5–14)	6 (3–11)	10 (6–16)
**BMI**^**b**^	22.5 (20.8–23.8)	27.3 (26.1–28.5)	32.9 (31.2–35.6)	26.3 (23.2–29.9)	29.0 (25.9–32.5)
**WC**^**b**^	–	–	–	–	100 (91–108)
**Age (in years)**^**b,d**^	36 (27–50)	51 (37–65)	55 (42–66)	46 (32–61)	59 (46–71)
**Sex**					
Men	527,253 (36.7)	707,939 (53.4)	394,948 (44.1)	1,630,140 (44.6)	137,298 (47.1)
Women	909,738 (63.3)	618,703 (46.6)	499,836 (55.9)	2,028,277 (55.4)	154,007 (52.9)
**MEDEA deprivation index**^**e**^					
Quintile 1	226,165 (15.7)	180,162 (13.6)	98,899 (11.0)	505,226 (13.8)	32,542 (11.2)
Quintile 2	208,133 (14.5)	189,510 (14.3)	119,236 (13.3)	516,879 (14.2)	39,826 (13.7)
Quintile 3	198,978 (13.9)	192,240 (14.5)	131,763 (14.7)	522,981 (14.3)	40,019 (13.7)
Quintile 4	193,565 (13.5)	195,463 (14.7)	142,024 (15.9)	531,052 (14.5)	41,767 (14.3)
Quintile 5	190,155 (13.2)	185,886 (14.0)	144,632 (16.2)	520,673 (14.2)	34,249 (11.8)
Rural	263,435 (18.3)	251,378 (18.9)	169,075 (18.9)	683,888 (18.7)	73,535 (25.2)
Missing	156,560 (10.9)	132,003 (10.0)	89,155 (10.0)	377,718 (10.3)	29,367 (10.1)
**Nationality (geographic region)**					
Spain	1,216,424 (84.6)	1,169,166 (88.1)	804,483 (89.9)	3,190,073 (87.2)	271,950 (93,3)
Europe (non-Spanish)	68,689 (4.8)	36,592 (2.8)	22,475 (2.5)	127,756 (3.5)	5560 (1.9)
Africa	15,968 (1.1)	13,208 (1.0)	6379 (0.7)	34,655 (1.0)	1019 (0.4)
America	75,117 (5.2)	63,293 (4.8)	37,867 (4.2)	176,277 (4.8)	7310 (2.5)
Asia	61,693 (4.3)	44,383 (3.3)	23,580 (2.7)	129,656 (3.5)	5466 (1.9)
**Smoking status**					
Never	674,872 (46.9)	688,304 (51.9)	487,643 (54.5)	1,850,819 (50.6)	174,775 (60.0)
Former	113,105 (7.9)	154,969 (11.7)	106,333 (11.9)	374,407 (10.2)	33,958 (11.6)
Current	438,103 (30.5)	308,376 (23.2)	177,332 (19.8)	923,811 (25.3)	58,468 (20.1)
Missing	210,911 (14.7)	174,993 (13.2)	123,476 (13.8)	509,380 (13.9)	24,104 (8.3)
**Alcohol intake**					
None	541,451 (37.7)	464,399 (35.0)	315,211 (35.2)	1,321,061 (36.1)	107,230 (36.8)
Low	340,721 (23.7)	325,238 (24.5)	173,382 (19.4)	839,341 (22.9)	60,568 (20.8)
High	25,114 (1.7)	28,074 (2.1)	19,043 (2.1)	72,231 (2.0)	7077 (2.4)
Missing	529,705 (36.9)	508,931 (38.4)	387,148 (43.3)	1,425,784 (39.0)	116,430 (40.0)
**Type 2 diabetes**	34,847 (2.4)	109,302 (8.2)	123,313 (13.8)	267,426 (7.3)	50,269 (17.3)
**Cause of end of follow-up**					
End of study	1,170,596 (81.5)	1,037,513 (78.2)	683,190 (76.4)	2,891,299 (79.0)	207,329 (71.2)
Cancer^f^	47,609 (3.3)	87,344 (6.6)	67,875 (7.6)	202,828 (5.6)	27,837 (9.5)
Death	51,777 (3.6)	82,920 (6.2)	70,090 (7.8)	204,787 (5.6)	33,702 (11.6)
Transferred-out	167,009 (11.6)	118,865 (9.0)	73,629 (8.2)	359,503 (9.8)	22,437 (7.7)

*BMI* body mass index, *MEDEA* “Mortalidad en áreas pequeñas Españolas y Desigualdades Socioeconómicas y Ambientales”, *WC* waist circumference

^a^Participants were followed from the study index date until cancer diagnosis, death, transferal out of the SIDIAP, or until the end of the study period (December 31, 2018)

^b^Median (interquartile range)

^c^Visits to general practitioners and nurses during the year of BMI assessment

^d^At baseline

^e^Quintile 1 represents the least deprived and quintile 5 represents the most deprived. Rural was included as a category since the index cannot be calculated for people living in rural areas

^f^Any, excl. non-melanoma skin cancer

### Non-linear BMI associations and analyses restricted to never smokers

**Fig. 2:**
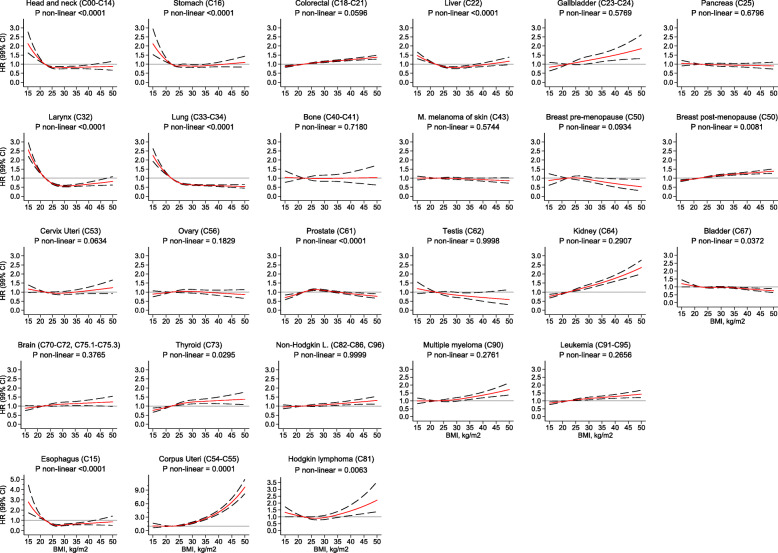
Association between body mass index and the risk of 26 cancer types in the overall population, allowing for non-linear effects, with 99% CIs. Notes: (1) The reference BMI for these plots was 22 kg/m^2^. Separate models were fitted for each cancer type and adjusted for smoking status, alcohol intake, nationality, the MEDEA deprivation index, type 2 diabetes, and had sex and age (5-year categories) in the strata statement. Each model had a restricted cubic spline for BMI with 3 knots placed at 21, 26, and 34 kg/m^2^ except for head and neck; stomach; trachea, bronchus, and lung; corpus uteri; and prostate and bladder cancers that had 5 knots placed at 19, 23, 26, 29, and 37 kg/m^2^. (2) Gallbladder includes biliary tract; lung includes trachea and bronchus; bone includes articular cartillage; brain includes the CNS, pituitary gland and pineal gland tumors. M. melanoma of skin stands for Malignant melanoma of skin; Non-Hodgkin L. stands for Non-Hodgkin lymphoma. (3) Models for ovary, cervix, and corpus uteri cancers were only computed in women, for breast pre-menopausal only in pre-menopausal women, for breast post-menopausal only in post-menopausal women, and for prostate and testis only computed in men. (4) All models have a scale up to a HR of 3 and are ordered by ascending ranking of ICD-10 codes, except for esophagus, corpus uteri, and Hodgkin lymphoma. Abbreviations: BMI, body mass index; CI, confidence interval; CNS, central nervous system; HR, hazard ratio; KG, kilograms; M, meters

**Fig. 3:**
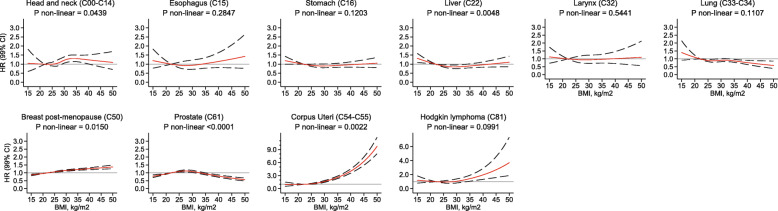
Association between body mass index and the risk of 10 cancer types in never smokers, allowing for non-linear effects, with 99% CIs. Notes: (1) The reference BMI for these plots was 22 kg/m^2^. Separate models were fitted for each cancer type and adjusted for alcohol intake, nationality, the MEDEA deprivation index, type 2 diabetes, and had sex and age (5-year categories) in the strata statement. Each model had a restricted cubic spline for BMI with 3 knots placed at 21, 26, and 34 kg/m^2^ except for head and neck, bronchus and lung, and corpus uteri that had 5 knots placed at 19, 23, 26, 29, and 37 kg/m^2^. (2) Lung includes trachea and bronchus tumors. (3) The association for corpus uteri cancer was only computed in women, for breast post-menopausal only in post-menopausal women, and for prostate cancer only in men. (4) All models have a scale up to a HR of 3, except for corpus uteri and Hodgkin lymphoma. Abbreviations: BMI, body mass index; CI, confidence interval; HR, hazard ratio; KG, kilograms; M, meters

BMI was non-linearly associated with ten of twenty-six cancer types (*p* for non-linearity < 0.01) (Fig. 2). For cancers of the head and neck, esophagus, stomach, larynx, trachea, bronchus, and lung, low BMI values were associated with a higher risk of these cancers. The risk stabilized above values of 22 kg/m^2^ (with HRs either at or below one). These non-linear relations disappeared when we restricted the analyses to never smokers (Fig. 3).

The curves for the associations between BMI and risk of cancers of the liver, breast post-menopausal, corpus uteri, prostate, and Hodgkin lymphoma were non-linear and were similarly shaped in the overall cohort and among never smokers (Figs. 2 and 3). Liver cancer showed an attenuated U-shaped curve, with a higher risk among participants with very low or very high BMI values. The risk of breast post-menopausal cancer seemed to increase linearly up to a BMI of 30 kg/m^2^, at which point the increase in risk diminished. For prostate cancer, the risk curve displayed an attenuated inverse U-shape, with a lower risk of cancer among those with low, normal, and very high BMIs, but an increased risk for those in the overweight range. For corpus uteri cancer, the risk increased faster than linear at higher BMI values. Finally, the association between BMI and Hodgkin lymphoma was J-shaped, with a modest higher risk of this lymphoma in people with low BMIs and a more markedly higher risk for those with high BMIs.

### Linear BMI associations and analyses restricted to never smokers

**Fig. 4:**
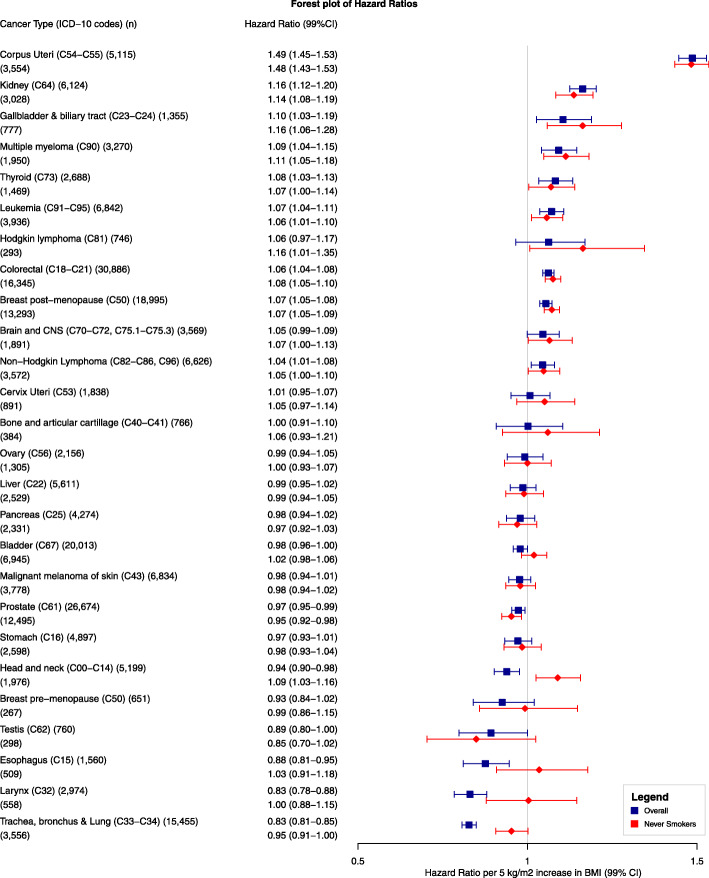
Forest plot of hazard ratios of 26 cancer types related to a linear increment in body mass index of 5 kg/m^2^ with 99% CIs, in the overall population, and in never smokers. Notes: (1) Separate models were fitted for each cancer type and adjusted for smoking status, alcohol intake, nationality, the MEDEA deprivation index, type 2 diabetes, and had sex and age (5-year categories) in the strata statement. (2) Cancer types are ordered by descending ranking. (3) Brain and CNS include pituitary gland and pineal gland tumors. (4) Models for ovary, cervix, and corpus uteri cancers were only computed in women, for breast pre-menopausal only in pre-menopausal women, for breast post-menopausal only in post-menopausal women, and for prostate and testis only computed in men. Abbreviations: BMI, body mass index; CI, confidence interval; CNS, central nervous system; KG, kilograms; M, meters; WC, waist circumference

A BMI increment of 5 kg/m^2^ (in multivariable analyses) was positively associated with risk of cancers of the corpus uteri (HR 1.49, 99%CI 1.45–1.53), kidney (1.16, 1.12–1.20), gallbladder and biliary tract (1.10, 1.03–1.19), multiple myeloma (1.09, 1.04–1.15), thyroid (1.08, 1.03–1.13), leukemia (1.07, 1.04–1.11), colorectal (1.06, 1.04–1.08), breast post-menopausal (1.07, 1.05–1.08), and non-Hodgkin lymphoma (1.04, 1.01–1.08) (Fig. 4). Results from the basic model are presented in Additional file 1: Table S3. Results for corpus uteri and breast-postmenopausal cancers should be interpreted in combination with the splines of Fig. 3 due to the evidence of non-linearity. For the five cancer types (trachea, bronchus and lung, larynx, esophagus, head and neck, and prostate) for which we observed an inverse association between BMI and cancer risk, there was evidence of non-linearity as shown in Fig. 3. After restricting the analyses to never smokers, BMI remained inversely associated only with risk of prostate cancer (0.95, 0.92–0.98), but became positively associated with risk of Hodgkin lymphoma (1.16, 1.01–1.35), and cancers of the head and neck (1.09, 1.03–1.16), and brain and CNS (1.07, 1.00–1.10).

### BMI and WC comparison in relation to cancer risk

Of the 291,305 participants who also had a WC assessment available, 27,837 were diagnosed with cancer from 2007 to 2018 (Table 1). Among eligible participants, the median follow-up time was 9.9 (IQR 7–12) years and the median age was 59 (IQR 46–71) years. The median WC was 100 (IQR 91–108) cm and the median BMI was 29 (IQR 26–33) kg/m^2^. Compared to the overall BMI cohort, these participants were older and had a higher median BMI and a higher prevalence of type 2 diabetes (Table 1).

**Fig. 5:**
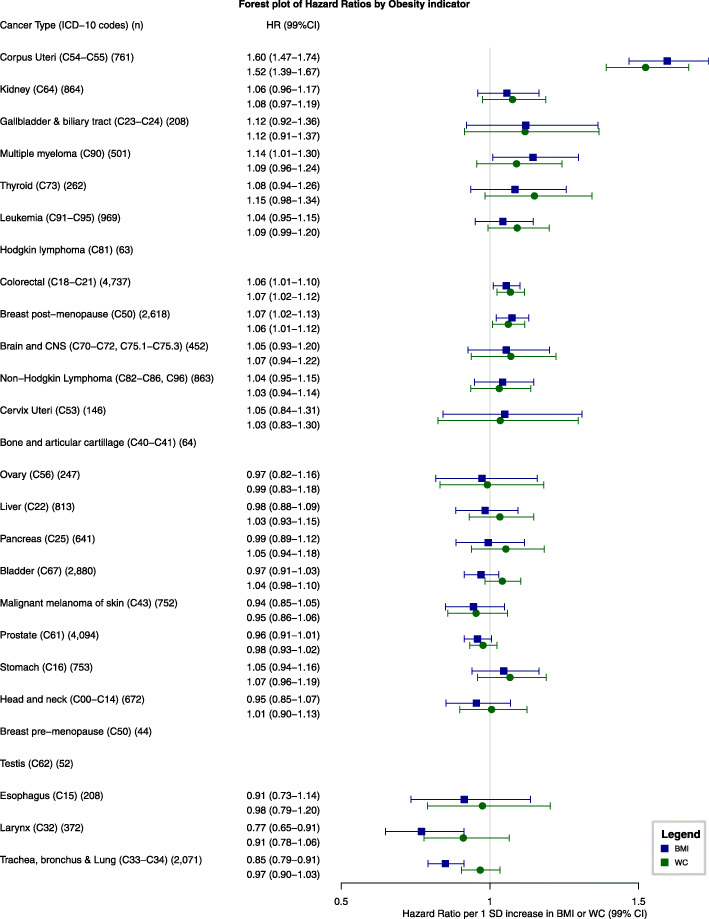
Forest plot of hazard ratios of 22 specific cancer sites related to a 1 standard deviation increase in body mass index and a 1 standard deviation increase in waist circumference. Notes: (1) SD for BMI and WC were 5.3 and 13.9 overall, 5.8 and 14.5 for women, 6.5 and 16.1 for pre-menopausal women, 5.4 and 13.3 for post-menopausal women, and 4.7 and 12.9 for men. (2) Separate models were fitted for each cancer type and adjusted for smoking status, alcohol intake, nationality, the MEDEA deprivation index, type 2 diabetes, and had sex and age (5-year categories) in the strata statement. (3) HRs are ordered by the descending ranking of BMI estimates from Fig. 4. (4) Brain and CNS include pituitary gland and pineal gland tumors. (5) Models for ovary, cervix, and corpus uteri cancers were only computed in women, for breast post-menopausal only in post-menopausal women, and for prostate only computed in men. (6) We only calculated hazard ratios for cancer types for which we ascertained at least 100 cancer cases. Abbreviations: BMI, body mass index; CI, confidence interval; CNS, central nervous system; SD, standard deviation; WC, waist circumference

We ascertained more than 100 cases for all cancers of interest except cancers of the bone and articular cartilage (64 cases), Hodgkin lymphoma (63), testis (52), and breast pre-menopausal (44) (Fig. 5). For all cancer sites, the 99% CIs of the HRs for WC (per 1 SD increase) and BMI overlapped. We observed the largest differences between the WC and BMI effect estimates for cancers of the bladder (HR for BMI 0.97, 99%CI 0.91–1.03; WC 1.04, 0.98–1.10), larynx (HR for BMI 0.77, 99%CI 0.65–0.91; WC 0.91, 0.78–1.06), and trachea, bronchus, and lung (HR for BMI 0.85, 99%CI 0.79–0.91; WC 0.97, 0.90–1.03), although the 99%CIs overlapped. Nonetheless, these results should be interpreted with caution due to evidence of non-linearity in the association between WC and risk of bladder and trachea, bronchus, and lung cancers (Additional file 1: Table S4).

### Sensitivity analyses

We assessed the robustness of our results by comparing the HRs of our main analyses to those from sensitivity analyses. We found that the HRs from our primary model (model 2) were similar to those from the sensitivity analyses. The CIs of the sensitivity analyses consistently included the main point estimate with only two exceptions (Additional file 1: Tables S5-S8). In the analysis in which we extended the minimum follow-up time from 1 to 4 years, the HRs from the main model for stomach and trachea, bronchus, and lung cancers (1-year follow-up) were not included in the CIs from the models with a 4-year minimum follow-up (stomach cancer with 1-year follow-up HR 0.99, 99%CI 0.99–1.00, vs. 4-year follow-up HR 1.01, 99%CI 1.00–1.01; trachea, bronchus, and lung cancer with 1-year follow-up HR 0.96, 99%CI 0.96–0.97, vs. 4-year follow-up HR 0.97, 99%CI 0.97–0.97; all HRs are per 1 kg/m^2^ increment in BMI) (Additional file 1: Table S5). We also re-ran the multivariable-adjusted models (model 2) using height on one hand and weight on the other as the main exposures (Additional file 1: Table S9). The nine cancer types that were positively associated with BMI were also all positively associated with weight, while six were so with height (colorectal, breast post-menopausal, kidney, thyroid, non-Hodgkin lymphoma, and leukemia). Corpus uteri cancer was negatively associated with height. The five cancer types for which we found a negative association with BMI were also negatively associated with weight while two of these were positively associated with height (trachea, bronchus, and lung and prostate cancers).

Furthermore, in the analysis comparing WC and BMI in relation to cancer risk, we assessed whether adding the residuals of the complementary adiposity indicator added valuable information to fully capture adiposity. This was not the case as the 99%CIs of the models comprising residuals always included the HRs from the main models (Additional file 1: Fig. S2). For example, for corpus uteri cancer, the model that only included BMI (HR 1.60, 99%CI 1.47–1.74) was similar to the one that included BMI and the residuals of WC (HR 1.61, 99%CI 1.48–1.76); the same was observed for the model that only included WC (HR 1.52, 99%CI 1.39–1.67) and the one that included WC and the residuals of BMI (HR 1.53, 99%CI 1.39–1.68). The CIs of the sensitivity analysis that further adjusted for height also consistently included the main point estimate of the main analyses comparing WC and BMI in relation to cancer risk (Additional file 1: Table S10).

## Discussion

### Main findings

In this prospective study that included 3,658,417 participants and 202,837 cancer cases, we found that a higher BMI was associated with risk of 18 of 26 cancer types, although these relations differed in terms of direction, shape, and smoking status at baseline. BMI was positively associated with risk of cancers of the *corpus uteri*, *kidney*, *gallbladder* *and biliary tract*, *thyroid*, *colorectum*, *breast post-menopausal*, *multiple myeloma*, *leukemia*, and *non-Hodgkin lymphoma* (in descending order of linear effect sizes). After restricting the analyses to never smokers to account for incomplete adjustment for smoking, BMI was also positively associated with *Hodgkin lymphoma* and cancers of the *head* *and* *neck*, and *brain* *and CNS*. BMI was associated in an inverse U-shaped manner with the risk of prostate cancer and in an L-shaped fashion with the risk of four cancers (*head and neck*, *esophagus*, *larynx*, and *trachea*, *bronchus, and* *lung*) in the overall cohort likely indicating residual confounding by smoking since the shape of these associations drastically changed among never smokers, except for prostate cancer.

In a subsample of 291,305 participants with a WC measurement and 27,837 cancer cases, we compared cancer risk estimates of WC and BMI. The 99% CIs of the WC and BMI effect estimates consistently overlapped, indicating that WC provides risk associations similar to BMI across a wide range of cancer types in our population.

### Strengths and limitations of this study

This study has several strengths. Firstly, to our knowledge, this is the first study to systematically compare both BMI and WC indicators in relation to the risk of a wide variety of cancers, including less frequently occurring ones. Secondly, owing to the large scale of the SIDIAP database, we were able to investigate the association between BMI and numerous cancer types in a Southern European region, increasing the external validity of results previously reported in Northwestern European countries [3, 4]. Lastly, we previously demonstrated the high quality of cancer diagnoses in the SIDIAP data and we conducted sensitivity analyses in regions where we could include cancer cases confirmed by population-based cancer registries (Additional file 1: Table S6) [18].

This study also has limitations. Firstly, the inclusion of individuals with a BMI measurement (62% of the SIDIAP adult population) could result in selection bias. However, the study participants were not substantially different from the overall SIDIAP population (Additional file 1: Table S2). Secondly, although we cannot exclude the possibility of exposure misclassification, we were empirically reassured that this was not a serious bias. The distribution of BMI in the SIDIAP was similar to population-based survey data and representative studies of the Spanish population (Additional file 1: Table S11). Thirdly, outcome misclassification could have biased our results towards the null because modest positive predictive values have been reported in a validation study of SIDIAP cancer diagnoses [18]. Fourth, residual confounding is an inherent limitation of observational studies; an example in our study was residual confounding for smoking status at baseline. Fifth, we did not have data on factors in the possible causal path between obesity and cancer, such as specific reproductive variables (e.g., parity, breastfeeding history), physical activity, and diet. Neither did we have information on cancer subtype or stage at diagnosis, which could have helped sharpen the analyses for certain cancers (e.g., *prostate* cancer). Fifth, while the magnitude of this study’s sample size has its advantages, some of the significant findings of this study could have been related to the large sample size. Another limitation was the missing covariate data which ranged from 10% (for the MEDEA deprivation index) to 39% (for alcohol intake risk). However, the results from our main analysis did not differ when we performed multiple imputations of these data (Additional file 1: Table S5). Finally, we had information for both BMI and WC for only 10% of the study participants. This limited our interpretation of the comparison of adiposity measures associated with cancer risk to individuals with both indicators and does not enable us to extrapolate the WC effect estimates to the general population.

### Interpretation and comparison with previous studies

The observed positive associations between BMI and different cancer types are in line with previous studies. The increased risk of breast post-menopausal and corpus uteri cancers has been consistently reported in the literature [25, 26]. Furthermore, our non-linear analyses showed that the higher the BMI, the greater the magnitude of risk of corpus uteri cancer which concurs with previous studies [4, 27]. The positive association between BMI and cancers of the colorectum, kidney, thyroid, and gallbladder and biliary tract is well recognized in the literature; however, nuances by subtype (kidney) [2, 28], histology (thyroid) [29], and sex (colorectal and gallbladder and biliary tract) have been reported [25, 30, 31]. In our data, we observed a stronger effect of BMI for gallbladder and biliary tract cancer in women and colorectal cancer in men, which is in line with previous studies (Additional file 1: Table S12) [25, 31]. Further, our results showed a clear pattern in the association between BMI and hematological cancers. The association observed between BMI and higher risk of leukemia and multiple myeloma has been consistently reported in the literature [25, 32–34], but the association between BMI and the lymphomas is less well established. Although our results for non-Hodgkin lymphoma are supported by two meta-analyses [25, 35], other studies have only reported a link with the subtype of diffuse large B cell lymphoma [36]. For Hodgkin lymphoma, we observed a J-shaped association with BMI, which concurs with a large study from the United Kingdom (UK) [37]. The positive association observed between BMI and cancers of the brain and CNS might have been driven by the inclusion of meningioma in this broad cancer group [2].

We also observed that the associations between BMI and respiratory tract cancers (head and neck, esophagus, larynx, and trachea, bronchus and lung) were L-shaped, suggesting that low BMIs are an approximation of heavy smoking. In the linear analyses restricted to never smokers, the associations between BMI and cancers of the larynx and esophagus became null, likely due to the opposite effects of BMI in adenoma and squamous cell carcinoma [25]. Also, among never smokers, BMI became positively associated with cancer of the head and neck and remained negatively associated with cancer of the trachea, bronchus, and lung, which concurs with other meta-analyses [25, 38–40]. For prostate cancer, we found an attenuated inverse U-shaped association which coincided with a large UK study [4]. The shape of this association could be explained by the dual effect of BMI on prostate cancer (inversely and positively associated with localized and advanced prostate cancer, respectively) [41]. Unfortunately, we did not have data on prostate cancer subtypes to test this hypothesis.

There were also differences between our results and those of previous studies. Despite the evidence supporting the inverse association between BMI and risk of breast pre-menopausal cancer [25], we observed a negative trend only with BMI values greater than 27 kg/m^2^. In addition, some studies described a positive association between BMI and cancers of the liver and stomach [42, 43]. Our results suggest these associations are non-linear and similarly shaped to a large UK study (U- and L-shaped for liver and stomach cancers, respectively) [4]. We noted that the non-linear association for stomach resembled the one for respiratory tract cancers, suggesting residual confounding by smoking status for this cancer as well.

In a post hoc analysis, modeling height and weight in mutually adjusted models, we found that the nine and five cancer types that were positively and negatively, respectively, associated with BMI (in linear models) were also all associated with weight in the same directions. On the other hand, height was positively associated with 14 cancer types (and only negatively associated with corpus uteri cancer) (Additional file 1: Table S9). This suggests that the associations observed for BMI (our main analysis) were driven by excess body weight rather than height. Height is a complex exposure and likely reflects the fact that more stem cells are at risk of acquiring driver mutations during cell division over time. A second possible explanation is that a common factor (such as insulin-like growth factor (IGF) 1) directly affects cancer risk as well as increasing height [44].

Finally, our results indicate that BMI and WC have a comparable relationship with cancer risk. The effect estimates of BMI and WC were similar although we observed moderate differences for cancers of the bladder, larynx, and trachea, bronchus, and lung. Contrarily to BMI, WC was not negatively associated with the risk of cancers of the larynx and trachea, bronchus, and lung. We hypothesized that this could be explained by smoking since smokers tend to have a higher WC, more visceral adipose tissue, and leaner body mass [5].

## Conclusion

In this large Southern European study, we found that a higher BMI was associated with higher risk of twelve cancer types. We provide novel evidence that higher BMI increases the risk of four hematological and head and neck (only among never smokers) cancers, and we confirmed associations reported in previous studies. Moreover, this study showed that BMI and WC result in comparable estimates of cancer risk associated with adiposity at a population level.

While the observational nature of this study prevents us from making policy and clinical recommendations, our findings reinforce the need for public health strategies focusing on the reduction of obesity for cancer prevention and indicate that assessing obesity-related cancer risk in primary care using BMI may be sufficient.

## Supplementary information

**Additional file 1: Appendix 1.** definition of menopause and use of hormonal replacement therapy variables. **Appendix 2.** STROBE Statement-Checklist. **Table S1.** diagnostic codes used to define cancer cases. **Table S2.** characteristics of individuals with and without a BMI recorded. **Table S3.** BMI-cancer risk associations: results of the basic adjustment models. **Table S4.** P for non-linearity in WC-cancer risk associations. **Table S5.** A wide range of sensitivity analyses of BMI-cancer risk associations. **Table S6.** Sensitivity analyses of BMI-cancer risk associations using cancer registry data to confirm SIDIAP cases. **Table S7.** Sensitivity analyses of BMI-cancer risk associations excluding subgroups of participants. **Table S8.** Sensitivity analyses of BMI-cancer risk associations for women only cancers. **Table S9.** Sensitivity analysis including results of BMI/height/weight-cancer risk associations. **Table S10.** Sensitivity analysis of BMI/WC-cancer risk associations including additional adjustment for height. **Table S11.** Comparison of BMI information recorded in the SIDIAP and other studies’ data. **Table S12.** BMI-cancer risk associations stratified by sex. Figure 1**.** Directed Acyclic Graph that guided our decisions in the control for confounding. Figure 2**.** Sensitivity analysis of BMI/WC-cancer risk associations, including mutual adjustment using residuals of BMI and WC.

## Data Availability

In accordance with current European and national law, the data used in this study is only available for the researchers participating in this project. Thus, we are not allowed to distribute or make publicly available the data to other parties. However, researchers from public institutions can request data from the SIDIAP and other sources (e.g., Cancer Registries) if they comply with certain requirements. Further information is available online (https://www.sidiap.org/index.php/menu-solicitudes-en/application-proccedure) or by contacting Anna Moleras (amoleras@idiapjgol.org).

## Abbreviations

BMI: Body mass index
CI: Confidence interval
CMBD: Minimum Basic DataSet
CNS: Central nervous system
GP: General practitioner
HR: Hazard ratio
HRT: Hormonal replacement therapy
ICD-9: International Classification for Diseases, 9th revision
ICD-10: International Classification for Diseases, 10th revision
MEDEA (deprivation index): “Mortalidad en áreas pequeñas Españolas y Desigualdades Socioeconómicas y Ambientales”
SD: Standard deviation
SIDIAP: Information System for Research in Primary Care
UK: United Kingdom
WC: Waist circumference
WHO: World Health Organization
