# Supplementary material for: Body mass index and waist circumference in relation to the risk of 26 types of cancer: a prospective cohort study of 3.5 million adults in Spain
Source: BMC Med. 2021 Jan 14;19:10. doi: 10.1186/s12916-020-01877-3 (PMC7807518; doi:10.1186/s12916-020-01877-3)
Supplement: Supplementary file 1 — Additional file 1: Appendix 1. definition of menopause and use of hormonal replacement therapy variables. Appendix 2. STROBE Statement-Checklist. Table S1. diagnostic codes used to define cancer cases. Table S2. characteristics of individuals with and without a BMI recorded. Table S3. BMI-cancer risk associations: results of the basic adjustment models. Table S4. P for non-linearity in WC-cancer risk associations. Table S5. A wide range of sensitivity analyses of BMI-cancer risk associations. Table S6. Sensitivity analyses of BMI-cancer risk associations using cancer registry data to confirm SIDIAP cases. Table S7. Sensitivity analyses of BMI-cancer risk associations excluding subgroups of participants. Table S8. Sensitivity analyses of BMI-cancer risk associations for women only cancers. Table S9. Sensitivity analysis including results of BMI/height/weight-cancer risk associations. Table S10. Sensitivity analysis of BMI/WC-cancer risk associations including additional adjustment for height. Table S11. Comparison of BMI information recorded in the SIDIAP and other studies’ data. Table S12. BMI-cancer risk associations stratified by sex. Figure 1. Directed Acyclic Graph that guided our decisions in the control for confounding. Figure 2. Sensitivity analysis of BMI/WC-cancer risk associations, including mutual adjustment using residuals of BMI and WC. [file 12916_2020_1877_MOESM1_ESM.docx]

**Additional file 1: BMC-Medicine**

**TITLE**

**Body mass index and waist circumference in relation to the risk of 26 types of cancer: a prospective cohort study of 3.5 million adults in Spain**

**AUTHORS**

**Martina Recalde, MPH^1,2^, Veronica Davila-Batista, PhD^1,3,4^, Yesika Díaz, BSc^1^, Michael Leitzmann, Professor^5^, Isabelle Romieu, Professor^6,7^, Heinz Freisling, PhD^3^*, Talita Duarte-Salles, PhD^1^***

**AFFILIATIONS**

1. Fundació Institut Universitari per a la recerca a l'Atenció Primària de Salut Jordi Gol i Gurina (IDIAPJGol), Gran Via Corts Catalanes, 587 àtic, 08007 Barcelona, Spain

2. Universitat Autònoma de Barcelona, Campus de Bellaterra, 08193 Bellaterra (Cerdanyola del Vallès), Spain

3. International Agency for Research on Cancer (IARC-WHO), 150 Cours Albert Thomas, 69008 Lyon, France

4. Consortium for Biomedical Research in Epidemiology and Public Health (CIBERESP), 28029 Madrid, Spain

5. Department of Epidemiology and Preventive Medicine, University of Regensburg, Franz-Josef-Strauss-Allee 11, 93053 Regensburg, Germany.

6. Center for Research on Population Health, National Institute of Public Health, Mexico City, Mexico.

7. Hubert Department of Global Health, Emory University, Atlanta, GA, USA.

*Joint last authorship

**Supplementary Material**

**Appendix S1**. Detailed definition of Menopause and use of Hormonal Replacement Therapy Variables.

**Table S1.** List of codes used to define cancer cases, according to the International Classification of Diseases, 10th and 9th edition.

**Figure S1.** Directed Acyclic Graph for the possible causal effect of obesity on cancer used to adjust the Cox proportional hazard models.

**Table S2.** Baseline characteristics of individuals in the SIDIAP database, by body mass index assessment.

**Table S3.** Association between body mass index and the risk of 26 cancer types: hazard ratios (99% CIs) in the basic and adjusted model.

**Table S4.** P for non-linearity of the models that investigate the risk of 22 cancer types associated to body mass index and waist circumference.

**Table S5.** Sensitivity Analyses: Association between body mass index and the risk of 26 cancer types: hazard ratios (99% CIs) across sensitivity analyses.

**Table S6.** Sensitivity Analyses: Hazard ratios of 15 cancer types related to body mass index, by cancer registry confirmation of SIDIAP cases.

**Table S7.** Sensitivity Analyses: Hazard ratios of 2 cancer types related to body mass index, excluding participants with history of chronic hepatitis b and c, and infection of Helicobacter Pylori.

**Table S8.** Sensitivity Analyses: Hazard ratios of 4 cancer types related to body mass index, restricting to post-menopausal women and adjusting for HRT use.

**Table S9.** Sensitivity Analysis: Hazard ratios (99% CIs) of 26 cancer types in relation to an increment in body mass index, height, or weight of 1 standard deviation.

**Figure S2.** Sensitivity Analyses: Forest plot of Hazard Ratios of 22 cancer types related to a lineal increment in body mass index (BMI) and waist circumference (WC) of 1 standard deviation, including mutual adjustment using residuals of BMI and WC.

**Table S10.** Sensitivity Analysis: Hazard Ratios of 22 cancer types related to a lineal increment in body mass index (BMI) and waist circumference (WC) of 1 standard deviation, including adjustment for height.

**Table S11.** Distribution of BMI in the SIDIAP database compared to population based-survey.

**Table S12.** Association between body mass index and the risk of 26 cancer types: hazard ratios (99% CIs), by sex.

**Appendix S2.** STROBE Statement-Checklist of items that should be included in reports of cohort studies.

**Appendix S1.** Detailed definition of Menopause and use of Hormonal Replacement Therapy Variables.

***Definition of menopause***

We considered a woman as menopausal if one of the following criteria was met (any time if previous to BMI assessment and up to 1 year after BMI assessment), in order of priority:

1. Menopause diagnosis (ICD-10 codes: N95*) while aged between ≥ 45 years and ≤ 55 years
2. “Age at menopause” registered in the “Atenció a la salut sexual i reproductiva” (ASSIR, Attention to sexual and reproductive health) centre while aged between ≥ 45 years and ≤ 55 years
3. “Menopause” registered in the ASSIR centre while aged between ≥ 45 years and ≤ 55 years
4. “Menopause year” registered in the ASSIR centre while aged between  ≥ 45 years and ≤ 55 years
5. Aged ≥ 50 years at the moment of BMI measurement

 Women who did not comply with these criteria were considered non-menopausal.

***Definition of Hormonal Replacement Therapy***

Women defined as menopausal with any pharmacy dispensation of the following drugs were considered under hormonal replacement therapy:

Abbreviations: ASSIR: Attention to sexual and reproductive health; BMI: Body Mass Index; ICD-10: International Classification for Diseases, 10th revision.

**Table S1.** List of codes used to define cancer cases, according to the International Classification of Diseases, 10th and 9th edition.

|  | **Codes** | |
| --- | --- | --- |
| **Cancer Type** | **ICD-10^a^** | **ICD-9^b^** |
| Head and neck | C00-C14 | 140-149 |
| Esophagus | C15 | 150 |
| Stomach | C16 | 151 |
| Colorectal | C18-C21 | 153, 154 |
| Liver | C-22 | 155 |
| Gallbladder & biliary tract | C23-C24 | 156 |
| Pancreas | C25 | 157 |
| Larynx | C32 | 161 |
| Trachea, bronchus & Lung | C33-C34 | 162 |
| Bone and articular cartilage | C40-C41 | 170 |
| Malignant melanoma of skin | C43 | 172 |
| Breast | C50 | 174, 175 |
| Cervix Uteri | C53 | 180 |
| Corpus Uteri | C54-C55 | 179, 182 |
| Ovary | C56 | 183, 183.0 |
| Prostate | C61 | 185 |
| Testis | C62 | 186 |
| Kidney | C64 | 189, 189.0 |
| Bladder | C67 | 188 |
| Brain and CNS^c^ | C70-C72, C75.1-C75.3 | 191, 192, 194.3, 194.4 |
| Thyroid | C73 | 193 |
| Hodgkin lymphoma | C81 | 201 |
| Non-Hodgkin Lymphoma | C82-C86, C96 | 200, 202 |
| Multiple myeloma | C90 | 203 |
| Leukemia | C91-C95 | 204-208 |

Notes: ^a^ ICD-10 is the classification system used in the SIDIAP. ^b^ ICD-9 is the classification system used in the hospital discharge database. ^c^ Include pituitary gland and pineal gland tumors.

Abbreviations: CNS: Central Nervous System; ICD-9: International Classification for Diseases; 9^th^ revision; ICD-10: International Classification for Diseases, 10^th^ revision; SIDIAP: Information System for Research in Primary Care.

**Figure S1.** Directed Acyclic Graph for the possible causal effect of obesity on cancer used to adjust the Cox proportional hazard models.


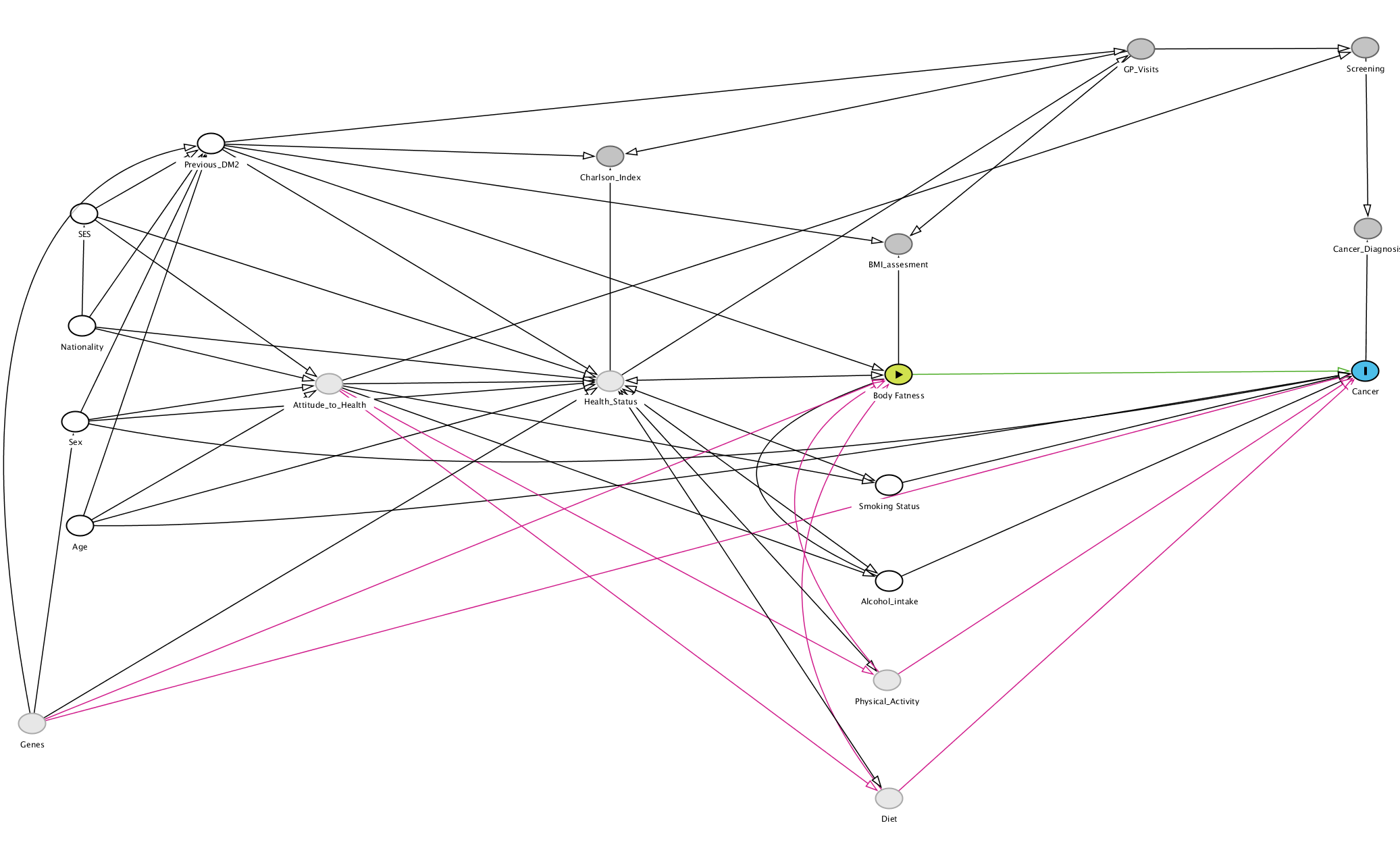


Abbreviations: BMI: Body Mass Index; DM2: Type 2 Diabetes Mellitus; GP: General Practitioner; SES: Socioeconomic Status.

**Table S2**. Baseline characteristics of individuals in the SIDIAP database, by body mass index assessment.

|  | **No BMI** | **With BMI** | **All** |
| --- | --- | --- | --- |
|  | 1,832,212 | 3,988,260 | 5,820,472 |
| **Sex** |  |  |  |
| Men | 1039565 (56.74%) | 1794506 (44.99%) | 2834071 (48.69%) |
| Women | 792647 (43.26%) | 2193754 (55.01%) | 2986401 (51.31%) |
| **Age^a^** | 32.79 (22.81-45.08) | 47.1 (33.14-62.61) | 41.76 (29.36-59.03) |
| **Age categories^a^** |  |  |  |
| 18-34 | 974771 (53.2%) | 1063338 (26.66%) | 2038109 (35.02%) |
| 35-49 | 485894 (26.52%) | 1058563 (26.54%) | 1544457 (26.53%) |
| 49-64 | 177074 (9.66%) | 952731 (23.89%) | 1129805 (19.41%) |
| ≥65 | 194473 (10.61%) | 913628 (22.91%) | 1108101 (19.04%) |
| **MEDEA Deprivation Index^b^** |  |  |  |
| Quintile 1 (Least Deprived) | 304714 (16.63%) | 560022 (14.04%) | 864736 (14.86%) |
| Quintile 2 | 257277 (14.04%) | 563379 (14.13%) | 820656 (14.1%) |
| Quintile 3 | 243374 (13.28%) | 567447 (14.23%) | 810821 (13.93%) |
| Quintile 4 | 232609 (12.7%) | 575340 (14.43%) | 807949 (13.88%) |
| Quintile 5 (Most Deprived) | 233671 (12.75%) | 562632 (14.11%) | 796303 (13.68%) |
| Rural | 289306 (15.79%) | 737786 (18.5%) | 1027092 (17.65%) |
| Missing | 271261 (14.81%) | 421654 (10.57%) | 692915 (11.9%) |
| **Smoking Status^c^** |  |  |  |
| Never | 574733 (31.37%) | 2339363 (58.66%) | 2914096 (50.07%) |
| Former | 59923 (3.27%) | 518042 (12.99%) | 577965 (9.93%) |
| Current | 295131 (16.11%) | 980939 (24.6%) | 1276070 (21.92%) |
| Missing | 902425 (49.25%) | 149916 (3.76%) | 1052341 (18.08%) |
| **Alcohol Intake^c^** |  |  |  |
| None | 354039 (19.32%) | 2135040 (53.53%) | 2489079 (42.76%) |
| Low | 202194 (11.04%) | 1298263 (32.55%) | 1500457 (25.78%) |
| High | 17139 (0.94%) | 102126 (2.56%) | 119265 (2.05%) |
| Missing | 1258840 (68.71%) | 452831 (11.35%) | 1711671 (29.41%) |
| **Visits to Health Centre^d^** | 1.88 (0.84-3.85) | 5.98 (3.07-10.71) | 4.35 (1.88-8.89) |
| **Type 2 Diabetes** | 25860 (1.41%) | 296222 (7.43%) | 322082 (5.53%) |
| **Hypertension** | 63958 (3.49%) | 570028 (14.29%) | 633986 (10.89%) |
| **Cause of exit** |  |  |  |
| End of study | 1202456 (65.63%) | 3175532 (79.62%) | 4377988 (75.22%) |
| Cancer | 22790 (1.24%) | 120046 (3.01%) | 142836 (2.45%) |
| Death | 132391 (7.23%) | 296143 (7.43%) | 428534 (7.36%) |
| Transferred-out | 474575 (25.9%) | 396539 (9.94%) | 871114 (14.97%) |

Notes: 1) The index date for participants with BMI information is the first BMI measurement between 18 and 100 years of age (excluding those who only have an assessment during pregnancy (first “remaining subjects” box in the flowchart of Figure 1) and for those without BMI information it is their first visit to a nurse or a GP. a. At baseline, in years; b. Quintile 1 represents the least deprived and quintile 5 represents the most deprived, Rural was included as a category since the index cannot be calculated for people living in rural areas; c. we consider any available information after index date; d. number of visits to the GP or nurse divided by time of follow-up.

Abbreviations: BMI: Body mass index; GP: General Practitioner; MEDEA: “Mortalidad en áreas pequeñas Españolas y Desigualdades Socioeconómicas y Ambientales”.

**Table S3.** Association between body mass index and the risk of 26 cancer types: hazard ratios (99% CIs) in the basic and adjusted model.

|  | **HR (99%CI) per 1 unit increase in BMI** | | |
| --- | --- | --- | --- |
| **Cancer Type** | **Cancer Cases** | **Basic (Model 1)** | **Adjusted (Model 2)** |
| Head and neck (C00-C14) | 5,199 | 0.98 (0.98-0.99) | 0.99 (0.98-0.99) |
| Esophagus (C15) | 1,560 | 0.97 (0.95-0.98) | 0.97 (0.96-0.99) |
| Stomach (C16) | 4,897 | 1.00 (0.99-1.00) | 0.99 (0.99-1.00) |
| Colorectal (C18-C21) | 30,886 | 1.01 (1.01-1.02) | 1.01 (1.01-1.01) |
| Liver (C22) | 5,611 | 1.00 (0.99-1.01) | 1.00 (0.99-1.00) |
| Gallbladder & biliary tract (C23-C24) | 1,355 | 1.02 (1.01-1.04) | 1.02 (1.01-1.03) |
| Pancreas (C25) | 4,274 | 1.00 (0.99-1.00) | 1.00 (0.99-1.00) |
| Larynx (C32) | 2,974 | 0.95 (0.94-0.96) | 0.96 (0.96-0.97) |
| Trachea, bronchus & Lung (C33-C34) | 15,455 | 0.95 (0.95-0.96) | 0.96 (0.96-0.97) |
| Bone and articular cartillage (C40-C41) | 766 | 1.00 (0.98-1.02) | 1.00 (0.99-1.02) |
| Malignant melanoma of skin (C43) | 6,834 | 0.99 (0.99-1.00) | 1.00 (0.99-1.00) |
| Breast pre-menopause (C50) | 651 | 0.98 (0.96-1.00) | 0.98 (0.97-1.00) |
| Breast post-menopause (C50) | 18,995 | 1.01 (1.01-1.01) | 1.01 (1.01-1.02) |
| Cervix Uteri (C53) | 1,838 | 1.00 (0.99-1.01) | 1.00 (0.99-1.01) |
| Corpus Uteri (C54-C55) | 5,115 | 1.09 (1.08-1.09) | 1.08 (1.08-1.09) |
| Ovary (C56) | 2,156 | 1.00 (0.99-1.01) | 1.00 (0.99-1.01) |
| Prostate (C61) | 26,674 | 0.99 (0.99-1.00) | 0.99 (0.99-1.00) |
| Testis (C62) | 760 | 0.98 (0.96-1.00) | 0.98 (0.96-0.99) |
| Kidney (C64) | 6,124 | 1.03 (1.02-1.04) | 1.03 (1.03-1.04) |
| Bladder (C67) | 20,013 | 0.99 (0.99-1.00) | 1.00 (0.99-1.00) |
| Brain and CNS (C70-C72, C75.1-C75.3) | 3,569 | 1.01 (1.00-1.02) | 1.01 (1.00-1.02) |
| Thyroid (C73) | 2,688 | 1.02 (1.01-1.03) | 1.02 (1.01-1.02) |
| Hodgkin lymphoma (C81) | 746 | 1.01 (0.99-1.03) | 1.01 (1.00-1.03) |
| Non-Hodgkin Lymphoma (C82-C86, C96) | 6,626 | 1.01 (1.00-1.02) | 1.01 (1.00-1.01) |
| Multiple myeloma (C90) | 3,270 | 1.02 (1.01-1.03) | 1.02 (1.01-1.03) |
| Leukemia (C91-C95) | 6,842 | 1.02 (1.01-1.02) | 1.01 (1.01-1.02) |

Notes: 1) The basic model (Model 1) only includes BMI as a linear term and sex and age (5-year categories) in the strata statement and the adjusted model (Model 2) which was used in our main analyses is adjusted for smoking status, alcohol intake, nationality, the MEDEA deprivation index, type 2 diabetes and has sex and age (5-year categories) in the strata statement. 2) Brain and CNS include pituitary gland and pineal gland tumors.

Abbreviations: BMI: Body Mass Index; CI: Confidence Interval; CNS: Central Nervous System; GP: General Practitioner; HR: Hazard Ratio.

**Table S4**. P for non-linearity of the models that investigate the risk of 22 cancer types associated to body mass index and waist circumference.

|  |  | **P for non-linearity** | |
| --- | --- | --- | --- |
| **Cancer Type** | **Cancer Cases** | **BMI** | **WC** |
| Head and neck (C00-C14) | 672 | 0.0124 | 0.9273 |
| Esophagus (C15) | 208 | 0.6397 | 0.9997 |
| Stomach (C16) | 753 | 0.0316 | 0.9348 |
| Colorectal (C18-C21) | 4,737 | 0.0042 | 0.1764 |
| Liver (C22) | 813 | <0.0001 | <0.0001 |
| Gallbladder & biliary tract (C23-C24) | 208 | 0.9319 | 0.9812 |
| Pancreas (C25) | 641 | 0.0202 | 0.3113 |
| Larynx (C32) | 372 | 0.0225 | 0.9435 |
| Trachea, bronchus & Lung (C33-C34) | 2,071 | 0.1658 | 0.0018 |
| Malignant melanoma of skin (C43) | 752 | 0.0524 | 0.2462 |
| Breast post-menopause (C50) | 2,618 | 0.6435 | 0.0168 |
| Cervix Uteri (C53) | 146 | 0.4081 | 0.6766 |
| Corpus Uteri (C54-C55) | 761 | 0.1878 | 0.0961 |
| Ovary (C56) | 247 | 0.8702 | 0.4294 |
| Prostate (C61) | 4,094 | 0.0210 | 0.0758 |
| Kidney (C64) | 864 | 0.9998 | 0.1376 |
| Bladder (C67) | 2880 | 0.2356 | 0.0067 |
| Brain and CNS (C70-C72, C75.1-C75.3) | 452 | 0.5698 | 0.6521 |
| Thyroid (C73) | 262 | 0.6861 | 0.9630 |
| Non-Hodgkin Lymphoma (C82-C86, C96) | 863 | 0.3979 | 0.0695 |
| Multiple myeloma (C90) | 501 | 0.5778 | 0.0058 |
| Leukemia (C91-C95) | 969 | 0.7661 | 0.2699 |

Notes: 1) Separate models were fitted for each cancer type and adjusted for smoking status, alcohol intake, nationality, the MEDEA deprivation index, type 2 diabetes and had sex and age (5-year categories) in the strata statement. 2) For each cancer type we compared the linear model for BMI/WC to the model had a restricted cubic spline for BMI/WC with 3 knots except for pancreas, corpus uteri and stomach cancers (BMI analyses) and corpus uteri and bladder (WC analyses) that had 5 knots using a likelihood-ratio test. 3) Brain and CNS include pituitary gland and pineal gland tumors. 4) Models for ovary, cervix and corpus uteri cancers were only computed in women, for breast post-menopausal only in post-menopausal women, and for prostate only computed in men. 5) We only analysed cancer types for which we ascertained at least 100 cancer cases.

Abbreviations: BMI: Body Mass Index; CNS: Central Nervous System; WC: Waist Circumference.

**Table S5**. Sensitivity Analyses: Association between body mass index and the risk of 26 cancer types: hazard ratios (99% CIs) across sensitivity analyses.

|  |  | **HR (99%CI) per 1 unit increase in BMI** | | | | | | |
| --- | --- | --- | --- | --- | --- | --- | --- | --- |
| **Cancer Type** | **Cancer Cases** | **Main model**  **(Model 2)** | **Model 2 + GP visits** | **Model 2+ Multiple Imputations** | **Cancer Cases** | **Model 2 + 2 years latency** | **Cancer Cases** | **Model 2+ 4 years latency** |
| Head and neck (C00-C14) | 5,199 | 0.99 (0.98-0.99) | 0.99 (0.98-0.99) | 0.99 (0.98-0.99) | 4,475 | 0.99 (0.98-0.99) | 3,104 | 0.99 (0.99-1.00) |
| Esophagus (C15) | 1,560 | 0.97 (0.96-0.99) | 0.97 (0.96-0.98) | 0.97 (0.96-0.98) | 1,401 | 0.98 (0.97-0.99) | 1,038 | 0.98 (0.96-0.99) |
| Stomach (C16) | 4,897 | 0.99 (0.99-1.00) | 0.99 (0.99-1.00) | 0.99 (0.99-0.99) | 4,342 | 1.00 (0.99-1.00) | 3,218 | 1.01 (1.00-1.01) |
| Colorectal (C18-C21) | 30,886 | 1.01 (1.01-1.01) | 1.01 (1.01-1.01) | 1.01 (1.01-1.01) | 27,463 | 1.01 (1.01-1.02) | 20,735 | 1.01 (1.01-1.02) |
| Liver (C22) | 5,611 | 1.00 (0.99-1.00) | 1.00 (0.99-1.00) | 0.99 (0.99-1.00) | 4,954 | 1.00 (0.99-1.01) | 3,672 | 1.00 (1.00-1.01) |
| Gallbladder & biliary tract (C23-C24) | 1,355 | 1.02 (1.01-1.03) | 1.02 (1.01-1.03) | 1.02 (1.01-1.03) | 1,208 | 1.02 (1.01-1.03) | 902 | 1.03 (1.01-1.04) |
| Pancreas (C25) | 4,274 | 1.00 (0.99-1.00) | 0.99 (0.99-1.00) | 0.99 (0.99-1.00) | 3,864 | 1.00 (1.00-1.01) | 3,062 | 1.00 (0.99-1.01) |
| Larynx (C32) | 2,974 | 0.96 (0.96-0.97) | 0.96 (0.95-0.97) | 0.96 (0.96-0.97) | 2,561 | 0.97 (0.96-0.97) | 1,791 | 0.97 (0.96-0.98) |
| Trachea, bronchus & Lung (C33-C34) | 15,455 | 0.96 (0.96-0.97) | 0.96 (0.96-0.97) | 0.96 (0.96-0.96) | 13,708 | 0.97 (0.96-0.97) | 10,301 | 0.97 (0.97-0.97) |
| Bone and articular cartillage (C40-C41) | 766 | 1.00 (0.99-1.02) | 1.00 (0.98-1.01) | 1.00 (0.99-1.01) | 667 | 0.99 (0.98-1.01) | 467 | 1.00 (0.98-1.01) |
| Malignant melanoma of skin (C43) | 6,834 | 1.00 (0.99-1.00) | 0.99 (0.99-1.00) | 1.00 (0.99-1.00) | 6,109 | 1.00 (0.99-1.00) | 4,499 | 1.00 (0.99-1.00) |
| Breast pre-menopause (C50) | 651 | 0.98 (0.97-1.00) | 0.98 (0.97-1.00) | 0.99 (0.98-0.99) | 521 | 0.99 (0.97-1.01) | 316 | 0.99 (0.97-1.01) |
| Breast post-menopause (C50) | 18,995 | 1.01 (1.01-1.02) | 1.01 (1.01-1.02) | 1.01 (1.01-1.02) | 23,291 | 1.01 (1.01-1.01) | 17,345 | 1.01 (1.01-1.02) |
| Cervix Uteri (C53) | 1,838 | 1.00 (0.99-1.01) | 1.00 (0.99-1.01) | 1.00 (0.99-1.01) | 1,570 | 1.00 (0.99-1.01) | 1,062 | 1.00 (0.99-1.01) |
| Corpus Uteri (C54-C55) | 5,115 | 1.08 (1.08-1.09) | 1.08 (1.08-1.09) | 1.08 (1.08-1.09) | 4,514 | 1.08 (1.08-1.09) | 3,346 | 1.09 (1.08-1.09) |
| Ovary (C56) | 2,156 | 1.00 (0.99-1.01) | 1.00 (0.99-1.01) | 0.99 (0.99-1.00) | 1,872 | 1.00 (0.99-1.01) | 1,370 | 0.99 (0.99-1.01) |
| Prostate (C61) | 26,674 | 0.99 (0.99-1.00) | 0.99 (0.99-1.00) | 0.99 (0.99-0.99) | 22,986 | 0.99 (0.99-1.00) | 16,254 | 0.99 (0.99-1.00) |
| Testis (C62) | 760 | 0.98 (0.96-0.99) | 0.98 (0.96-0.99) | 0.98 (0.96-0.99) | 648 | 0.98 (0.96-1.00) | 459 | 0.98 (0.96-1.00) |
| Kidney (C64) | 6,124 | 1.03 (1.03-1.04) | 1.03 (1.02-1.03) | 1.03 (1.03-1.04) | 5,437 | 1.03 (1.03-1.04) | 4,191 | 1.03 (1.03-1.04) |
| Bladder (C67) | 20,013 | 1.00 (0.99-1.00) | 0.99 (0.99-1.00) | 1.00 (0.99-1.00) | 17,616 | 1.00 (0.99-1.00) | 12,964 | 1.00 (0.99-1.00) |
| Brain and CNS (C70-C72, C75.1-C75.3) | 3,569 | 1.01 (1.00-1.02) | 1.01 (1.00-1.01) | 1.01 (1.00-1.01) | 3,097 | 1.01 (1.00-1.01) | 2,267 | 1.01 (1.00-1.02) |
| Thyroid (C73) | 2,688 | 1.02 (1.01-1.02) | 1.01 (1.01-1.02) | 1.02 (1.01-1.02) | 2,338 | 1.02 (1.01-1.02) | 1,708 | 1.02 (1.01-1.03) |
| Hodgkin lymphoma (C81) | 746 | 1.01 (1.00-1.03) | 1.01 (1.00 -1.03) | 1.01 (1.00-1.02) | 625 | 1.01 (0.99-1.02) | 425 | 1.02 (1.00-1.04) |
| Non-Hodgkin Lymphoma (C82-C86, C96) | 6,626 | 1.01 (1.00-1.01) | 1.01 (1.00-1.01) | 1.01 (1.00-1.01) | 5,862 | 1.01 (1.01-1.02) | 4,447 | 1.01 (1.01-1.02) |
| Multiple myeloma (C90) | 3,270 | 1.02 (1.01-1.03) | 1.02 (1.01-1.02) | 1.02 (1.01-1.02) | 2,887 | 1.02 (1.01-1.03) | 2,165 | 1.02 (1.01-1.03) |
| Leukemia (C91-C95) | 6,842 | 1.01 (1.01-1.02) | 1.01 (1.01-1.02) | 1.01 (1.01-1.02) | 6,071 | 1.01 (1.01-1.02) | 4,514 | 1.02 (1.01-1.02) |

Notes: 1) The adjusted model (Model 2) which was used in our main analyses is adjusted for smoking status, alcohol intake, nationality, the MEDEA deprivation index, type 2 diabetes and has sex and age (5-year categories) in the strata statement. 2) “Model 2 + GP visits” is also adjusted for the number of GP consultations at year of study entry. 3) “Model 2 + Multiple imputations” corresponds to Model 2 using multiple imputations for covariates with missing values. 4) “Model 2 + 2 years latency” and “Model 2 + 4 years latency” extend the latency period to 2 and 4 years. 5) Brain and CNS include pituitary gland and pineal gland tumors.

Abbreviations: BMI: Body Mass Index; CI: Confidence Interval; CNS: Central Nervous System; GP: General Practitioner; HR: Hazard Ratio.

**Table S6.** Sensitivity Analyses: Hazard ratios of 15 cancer types related to body mass index, by cancer registry confirmation of SIDIAP cases.

|  | **HR (99%CI) per 1 unit increase in BMI** | | | | | |
| --- | --- | --- | --- | --- | --- | --- |
|  | **SIDIAP (n=3,658,417)** | | **Catchment area of the Girona, Tarragona and Hospital del Mar cancer registries (n=323,325)** | | | |
| **Cancer Type** | **Cancer cases** | **Adjusted (Model 2)** | **Cancer Cases** | **Adjusted (Model 2)** | **Cancer Cases** | **Adjusted (Model 2) with cases confirmed by the registries** |
| Head and neck (C00-C14) | 5,199 | 0.99 (0.98-0.99) | 365 | 1.00 (0.98-1.02) | 91 | 0.98 (0.94-1.03) |
| Esophagus (C15) | 1,560 | 0.97 (0.96-0.99) | 127 | 0.94 (0.90-0.98) | 37 | - |
| Stomach (C16) | 4,897 | 0.99 (0.99-1.00) | 239 | 0.99 (0.96-1.02) | 88 | 0.96 (0.92-1.01) |
| Colorectal (C18-C21) | 30,886 | 1.01 (1.01-1.01) | 1,749 | 1.01 (1.00-1.02) | 643 | 1.01 (1.00-1.03) |
| Liver (C22) | 5,611 | 1.00 (0.99-1.00) | 293 | 0.98 (0.96-1.01) | 75 | 0.94 (0.89-0.99) |
| Gallbladder & biliary tract (C23-C24) | 1,355 | 1.02 (1.01-1.03) | 60 | 0.97 (0.92-1.03) | 11 | - |
| Pancreas (C25) | 4,274 | 1.00 (0.99-1.00) | 209 | 0.99 (0.96-1.02) | 44 | - |
| Larynx (C32) | 2,974 | 0.96 (0.96-0.97) | 203 | 0.97 (0.94-1.00) | 69 | 0.98 (0.93-1.04) |
| Trachea, bronchus & Lung (C33-C34) | 15,455 | 0.96 (0.96-0.97) | 866 | 0.94 (0.92-0.96) | 305 | 0.93 (0.91-0.96) |
| Bone and articular cartillage (C40-C41) | 766 | 1.00 (0.99-1.02) | 58 | - | 4 | - |
| Malignant melanoma of skin (C43) | 6,834 | 1.00 (0.99-1.00) | 483 | 1.00 (0.98-1.02) | 103 | 0.99 (0.95-1.03) |
| Breast pre-menopause (C50) | 651 | 0.98 (0.97-1.00) | 37 | - | 23 | - |
| Breast post-menopause (C50) | 18,995 | 1.01 (1.01-1.02) | 1,096 | 1.02 (1.01-1.03) | 439 | 1.01 (1.00-1.03) |
| Cervix Uteri (C53) | 1,838 | 1.00 (0.99-1.01) | 152 | 1.01 (0.98-1.04) | 46 | - |
| Corpus Uteri (C54-C55) | 5,115 | 1.08 (1.08-1.09) | 304 | 1.09 (1.08-1.11) | 119 | 1.10 (1.07-1.13) |
| Ovary (C56) | 2,156 | 1.00 (0.99-1.01) | 141 | 0.99 (0.97-1.03) | 31 | - |
| Prostate (C61) | 26,674 | 0.99 (0.99-1.00) | 1,457 | 0.99 (0.98-1.00) | 584 | 0.99 (0.97-1.01) |
| Testis (C62) | 760 | 0.98 (0.96-0.99) | 75 | 1.02 (0.98-1.07) | 25 | - |
| Kidney (C64) | 6,124 | 1.03 (1.03-1.04) | 399 | 1.04 (1.02-1.06) | 114 | 1.05 (1.01-1.09) |
| Bladder (C67) | 20,013 | 1.00 (0.99-1.00) | 1,118 | 1.01 (0.99-1.02) | 281 | 1.02 (0.99-1.05) |
| Brain and CNS (C70-C72, C75.1-C75.3) | 3,569 | 1.01 (1.00-1.02) | 198 | 0.98 (0.95-1.01) | 41 | - |
| Thyroid (C73) | 2,688 | 1.02 (1.01-1.02) | 235 | 1.02 (1.00-1.05) | 80 | 1.02 (0.98-1.06) |
| Hodgkin lymphoma (C81) | 746 | 1.01 (1.00-1.03) | 44 | - | 16 | - |
| Non-Hodgkin Lymphoma (C82-C86, C96) | 6,626 | 1.01 (1.00-1.01) | 315 | 0.99 (0.97-1.02) | 87 | 0.99 (0.94-1.04) |
| Multiple myeloma (C90) | 3,270 | 1.02 (1.01-1.03) | 109 | 1.02 (0.98-1.06) | 26 | - |
| Leukemia (C91-C95) | 6,842 | 1.01 (1.01-1.02) | 253 | 1.02 (1.00-1.05) | 67 | 1.05 (1.00-1.10) |

Notes: 1) The adjusted model (Model 2 in SIDIAP) which was used in our main analyses is adjusted for smoking status, alcohol intake, nationality, the MEDEA deprivation index, type 2 diabetes and has sex and age (5-year categories) in the strata statement. 2) Model 2 in the catchment area of the cancer registries was only run among individuals that belong to the catchment area of the population based Cancer Registries of the provinces of Girona and Tarragona (data available from 2008 until 2015 for Girona and from 2008 until 2013 for Tarragona), and the Hospital Registry of the Hospital del Mar in Barcelona (data available from 2008 until 2016). 3) “Adjusted (Model 2) with cases confirmed by the registries” only uses as outcomes the SIDIAP cancer cases confirmed by the cancer registries. 4) For cancers of the Hodgkin lymphoma, non-Hodgkin lymphoma, multiple myeloma and leukemia we did not have data from the Hospital del Mar. 5) We only calculated hazard ratios for cancer types for which we ascertained at least 50 cancer cases. 6) Brain and CNS include pituitary gland and pineal gland tumors.

Abbreviations: BMI: Body Mass Index; CI: Confidence Interval; CNS: Central Nervous System; HR: Hazard Ratio; SIDIAP: Information System for Research in Primary Care.

**Table S7.** Sensitivity Analyses: Hazard ratios of 2 cancer types related to body mass index, excluding participants with history of chronic Hepatitis b and c, and infection of Helicobacter Pylori.

|  |  |  | **HR (99%CI) per 1 unit increase in BMI** | | | |
| --- | --- | --- | --- | --- | --- | --- |
| **Cancer Type** | **Reason for exclusion** | **Excluded Participants** | **Cancer Cases** | **Adjusted (Model 2)** | **Cancer Cases** | **Adjusted (Model 2)  Excluding participants** |
| Stomach (C16) | Helicobacter Pylori Infection | 32,961 | 4,897 | 0.99 (0.99-1.00) | 4,895 | 0.99 (0.99-1.00) |
| Liver (C22) | Chronic Hepatitis B or C | 46,416 | 5,611 | 1.00 (0.99-1.00) | 5,576 | 1.00 (0.99-1.00) |

Notes: 1) The adjusted model (Model 2 in SIDIAP) which was used in our main analyses is adjusted for smoking status, alcohol intake, nationality, the MEDEA deprivation index, type 2 diabetes and has sex and age (5-year categories) in the strata statement. 2) “Adjusted (Model 2) excluding participants” corresponds to Model 2 but excludes excluding participants with history of chronic Hepatitis b and c and infection of Helicobacter Pylori (Adjusted excluding participants).

Abbreviations: BMI: Body Mass Index; CI: Confidence Interval; HR: Hazard Ratio.

**Table S8.** Sensitivity Analyses: Hazard ratios of 4 cancer types related to body mass index, restricting to post-menopausal women and adjusting for HRT use.

|  | **HR (99%CI) per 1 unit increase in BMI** | | | | |
| --- | --- | --- | --- | --- | --- |
| **Cancer Type** | **Cancer cases** | **Adjusted (Model 2)** | **Cancer cases** | **Model 2 in post-menopausal women** | **Model 2 in post-menopausal women + HRT adjustment** |
| Breast post-menopause (C50) | 18,995 | 1.01 (1.01-1.02) | 18,995 | - 1. (1.01-1.02) | 1.01 (1.01-1.02) |
| Cervix Uteri (C53) | 1,838 | 1.00 (0.99-1.01) | 971 | 1.01 (1.00-1.02) | 1.01 (1.00-1.02) |
| Corpus Uteri (C54-C55) | 5,115 | 1.08 (1.08-1.09) | 4,295 | 1.09 (1.08-1.09) | 1.08 (1.08-1.09) |
| Ovary (C56) | 2,156 | 1.00 (0.99-1.01) | 1,473 | 0.99 (0.98-1.00) | 0.99 (0.98-1.00) |

Notes: 1) The adjusted model (Model 2 in SIDIAP) which was used in our main analyses is adjusted for smoking status, alcohol intake, nationality, the MEDEA deprivation index, type 2 diabetes and has sex and age (5-year categories) in the strata statement. 2) “Model 2 in post-menopausal women” was only run among post-menopausal women. 3) “Model 2 in post-menopausal women + HRT adjustment” was only run among post-menopausal women and also adjusted for HRT use. 4) The menopause and HRT definitions can be consulted in Appendix S1.

Abbreviations: BMI: Body Mass Index; CI: Confidence Interval; HR: Hazard Ratio; HRT: Hormonal replacement therapy.

**Table S9.** Sensitivity Analysis: Hazard ratios (99% CIs) of 26 cancer types in relation to an increment in body mass index, height, or weight of 1 standard deviation.

|  | **HR (99%CI) per one SD increment in BMI, height or weight** | | | |
| --- | --- | --- | --- | --- |
| **Cancer Type** | **Cancer Cases** | **BMI (Model 2)** | **Height (Model 2)** | **Weight (Model 2)** |
| Head and neck (C00-C14) | 5,199 | 0.94 (0.90-0.98) | 0.95 (0.89-1.01) | 0.91 (0.87-0.96) |
| Esophagus (C15) | 1,560 | 0.87 (0.80-0.94) | 1.03 (0.92-1.14) | 0.86 (0.79-0.94) |
| Stomach (C16) | 4,897 | 0.97 (0.93-1.01) | 0.93 (0.88-1.00) | 0.96 (0.91-1.01) |
| Colorectal (C18-C21) | 30,886 | 1.06 (1.05-1.08) | 1.05 (1.03-1.08) | 1.09 (1.07-1.11) |
| Liver (C22) | 5,611 | 0.99 (0.95-1.03) | 0.96 (0.91-1.02) | 0.97 (0.93-1.02) |
| Gallbladder & biliary tract (C23-C24) | 1,355 | 1.11 (1.03-1.20) | 0.89 (0.79-1.01) | 1.13 (1.03-1.24) |
| Pancreas (C25) | 4,274 | 0.98 (0.94-1.02) | 1.10 (1.03-1.17) | 0.98 (0.92-1.03) |
| Larynx (C32) | 2,974 | 0.82 (0.78-0.87) | 1.06 (0.98-1.15) | 0.80 (0.75-0.86) |
| Trachea, bronchus & Lung (C33-C34) | 15,455 | 0.82 (0.80-0.84) | 1.19 (1.15-1.23) | 0.80 (0.78-0.83) |
| Bone and articular cartillage (C40-C41) | 766 | 1.00 (0.90-1.11) | 1.19 (1.02-1.38) | 1.00 (0.89-1.13) |
| Malignant melanoma of skin (C43) | 6,834 | 0.98 (0.94-1.01) | 1.25 (1.19-1.32) | 0.99 (0.95-1.03) |
| Breast pre-menopause (C50) | 651 | 0.92 (0.83-1.02) | 1.04 (0.93-1.16) | 0.93 (0.83-1.03) |
| Breast post-menopause (C50) | 18,995 | 1.07 (1.05-1.09) | 1.13 (1.10-1.15) | 1.09 (1.07-1.11) |
| Cervix Uteri (C53) | 1,838 | 1.01 (0.95-1.08) | 0.98 (0.91-1.05) | 1.02 (0.95-1.08) |
| Corpus Uteri (C54-C55) | 5,115 | 1.57 (1.52-1.62) | 0.89 (0.85-0.93) | 1.59 (1.54-1.64) |
| Ovary (C56) | 2,156 | 0.99 (0.93-1.05) | 1.05 (0.98-1.13) | 1.00 (0.94-1.06) |
| Prostate (C61) | 26,674 | 0.98 (0.96-0.99) | 1.04 (1.02-1.06) | 0.98 (0.96-1.00) |
| Testis (C62) | 760 | 0.90 (0.81-1.00) | 1.25 (1.12-1.41) | 0.90 (0.81-1.01) |
| Kidney (C64) | 6,124 | 1.17 (1.13-1.21) | 1.10 (1.04-1.16) | 1.22 (1.17-1.26) |
| Bladder (C67) | 20,013 | 0.98 (0.96-1.00) | 1.10 (1.06-1.13) | 0.99 (0.96-1.01) |
| Brain and CNS (C70-C72, C75.1-C75.3) | 3,569 | 1.05 (1.00-1.10) | 1.09 (1.01-1.17) | 1.06 (1.00-1.12) |
| Thyroid (C73) | 2,688 | 1.09 (1.03-1.14) | 1.21 (1.12-1.32) | 1.13 (1.07-1.20) |
| Hodgkin lymphoma (C81) | 746 | 1.07 (0.96-1.18) | 1.04 (0.89-1.22) | 1.08 (0.96-1.21) |
| Non-Hodgkin Lymphoma (C82-C86, C96) | 6,626 | 1.05 (1.01-1.08) | 1.12 (1.06-1.18) | 1.07 (1.03-1.11) |
| Multiple myeloma (C90) | 3,270 | 1.10 (1.04-1.15) | 1.01 (0.94-1.09) | 1.13 (1.06-1.20) |
| Leukemia (C91-C95) | 6,842 | 1.07 (1.04-1.11) | 1.07 (1.01-1.13) | 1.10 (1.06-1.15) |

Notes: 1) The BMI (Model 2) was used in our main analyses and is adjusted for smoking status, alcohol intake, nationality, the MEDEA deprivation index, type 2 diabetes and has sex and age (5-year categories) in the strata statement. 2) The Height (Model 2) includes height as the main exposure and is adjusted for weight, smoking status, alcohol intake, nationality, the MEDEA deprivation index, type 2 diabetes and has sex and age (5-year categories) in the strata statement. 3) The Weight (Model 2) includes weight as the main exposure and is adjusted for height, smoking status, alcohol intake, nationality, the MEDEA deprivation index, type 2 diabetes and has sex and age (5-year categories) in the strata statement. 4) Models for ovary, cervix and corpus uteri cancers were only computed in women, for breast pre-menopausal only in pre-menopausal women, for breast post-menopausal only in post-menopausal women, and for prostate and testis only in men. 5) SD for BMI, height and weight were 5.23, 9.95 and 15.71 overall, 5.68, 7.38 and 13.85 for women, 5.33, 6.59 and 14.05 for pre-menopausal women, 5.38, 6.81 and 13.22 for post-menopausal women; and 4.59, 7.80 and 14.54 for men. 6) Brain and CNS include pituitary gland and pineal gland tumors.

Abbreviations: BMI: Body Mass Index; CI: Confidence Interval; CNS: Central Nervous System; HR: Hazard Ratio, SD: Standard Deviation,

**Figure S2.** Sensitivity Analysis: Forest plot of Hazard Ratios of 22 cancer types related to a lineal increment in body mass index (BMI) and waist circumference (WC) of 1 standard deviation, including mutual adjustment using residuals of BMI and WC.

**
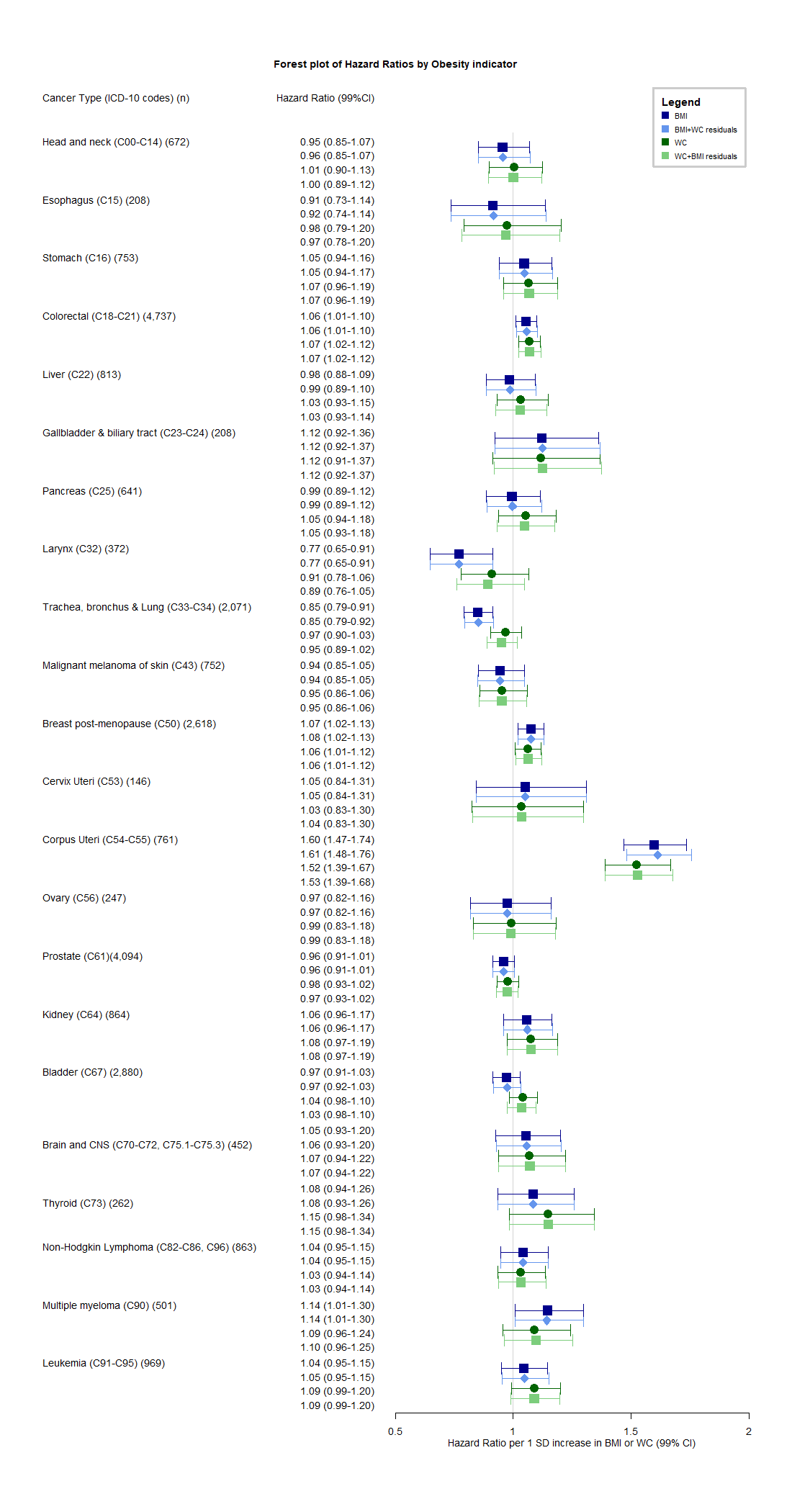
**

Notes: 1) SD for BMI and WC were 5.3 and 13.9 overall, 5.8 and 14.5 for women, 5.4 and 13.3 for post-menopausal women and 4.7 and 12.9 for men. 2) Separate models were fitted for each cancer type and adjusted for smoking status, alcohol intake, nationality, the MEDEA deprivation index, type 2 diabetes and had sex and age (5-year categories) in the strata statement. 3) “BMI + WC residuals” and “WC + BMI residuals” are models also adjusted by the residuals of the complementary adiposity indicator. 3) HRs are ordered by the descending ranking of BMI from main Figure 2. 4) Brain and CNS include pituitary gland and pineal gland tumors. 5) Models for ovary, cervix and corpus uteri cancers were only computed in women, for breast post-menopausal only in post-menopausal women, and for prostate only computed in men. 6) We only calculated hazard ratios for cancer types for which we ascertained at least 100 cancer cases. Abbreviations: BMI: Body Mass Index; CI: Confidence Interval; CNS: Central Nervous System; SD: Standard Deviation; WC: Waist Circumference.

**Table S10.** Sensitivity Analysis: Hazard Ratios of 22 cancer types related to a lineal increment in body mass index (BMI) and waist circumference (WC) of 1 standard deviation, including adjustment for height.

|  | **HR (99%CI) per 1 standard deviation increase in BMI and WC** | | | | |
| --- | --- | --- | --- | --- | --- |
| **Cancer Type** | **Cancer Cases** | **Main model**  **for BMI** | **Main model**  **for BMI + Height** | **Main model**  **for WC** | **Main model**  **For WC + Height** |
| Head and neck (C00-C14) | 672 | 0.95 (0.85-1.07) | 0.95 (0.85-1.07) | 1.01 (0.90-1.13) | 1.00 (0.90-1.13) |
| Esophagus (C15) | 208 | 0.91 (0.73-1.14) | 0.91 (0.73-1.14) | 0.98 (0.79-1.20) | 1.00 (0.81-1.24) |
| Stomach (C16) | 753 | 1.05 (0.94-1.16) | 1.04 (0.93-1.16) | 1.07 (0.96-1.19) | 1.06 (0.95-1.19) |
| Colorectal (C18-C21) | 4,737 | 1.06 (1.01-1.10) | 1.06 (1.01-1.11) | 1.07 (1.02-1.12) | 1.06 (1.02-1.11) |
| Liver (C22) | 813 | 0.98 (0.88-1.09) | 0.98 (0.88-1.09) | 1.03 (0.93-1.15) | 1.04 (0.94-1.16) |
| Gallbladder & biliary tract (C23-C24) | 208 | 1.12 (0.92-1.36) | 1.11 (0.90-1.36) | 1.12 (0.91-1.37) | 1.11 (0.90-1.36) |
| Pancreas (C25) | 641 | 0.99 (0.89-1.12) | 1.01 (0.90-1.14) | 1.05 (0.94-1.18) | 1.04 (0.92-1.17) |
| Larynx (C32) | 372 | 0.77 (0.65-0.91) | 0.76 (0.64-0.90) | 0.91 (0.78-1.06) | 0.92 (0.78-1.07) |
| Trachea, bronchus & Lung (C33-C34) | 2,071 | 0.85 (0.79-0.91) | 0.85 (0.79-0.91) | 0.97 (0.90-1.03) | 0.96 (0.90-1.03) |
| Malignant melanoma of skin (C43) | 752 | 0.94 (0.85-1.05) | 0.96 (0.86-1.06) | 0.95 (0.86-1.06) | 0.94 (0.84-1.04) |
| Breast post-menopause (C50) | 2,618 | 1.07 (1.02-1.13) | 1.10 (1.04-1.16) | 1.06 (1.01-1.12) | 1.06 (1.01-1.12) |
| Cervix Uteri (C53) | 146 | 1.05 (0.84-1.31) | 1.07 (0.85-1.33) | 1.03 (0.83-1.30) | 1.04 (0.82-1.30) |
| Corpus Uteri (C54-C55) | 761 | 1.60 (1.47-1.74) | 1.63 (1.49-1.77) | 1.52 (1.39-1.67) | 1.54 (1.40-1.69) |
| Ovary (C56) | 247 | 0.97 (0.82-1.16) | 0.95 (0.80-1.14) | 0.99 (0.83-1.18) | 0.99 (0.83-1.18) |
| Prostate (C61) | 4,094 | 0.96 (0.91-1.01) | 0.96 (0.92-1.01) | 0.98 (0.93-1.02) | 0.98 (0.93-1.02) |
| Kidney (C64) | 864 | 1.06 (0.96-1.17) | 1.07 (0.97-1.18) | 1.08 (0.97-1.19) | 1.06 (0.96-1.17) |
| Bladder (C67) | 2,880 | 0.97 (0.91-1.03) | 0.97 (0.92-1.04) | 1.04 (0.98-1.10) | 1.03 (0.97-1.09) |
| Brain and CNS (C70-C72, C75.1-C75.3) | 452 | 1.05 (0.93-1.20) | 1.06 (0.93-1.21) | 1.07 (0.94-1.22) | 1.08 (0.94-1.23) |
| Thyroid (C73) | 262 | 1.08 (0.94-1.26) | 1.10 (0.95-1.28) | 1.15 (0.98-1.34) | 1.15 (0.98-1.34) |
| Non-Hodgkin Lymphoma (C82-C86, C96) | 863 | 1.04 (0.95-1.15) | 1.06 (0.96-1.17) | 1.03 (0.94-1.14) | 1.02 (0.92-1.13) |
| Multiple myeloma (C90) | 501 | 1.14 (1.01-1.30) | 1.16 (1.02-1.32) | 1.09 (0.96-1.24) | 1.09 (0.96-1.25) |
| Leukemia (C91-C95) | 969 | 1.04 (0.95-1.15) | 1.06 (0.97-1.17) | 1.09 (0.99-1.20) | 1.08 (0.99-1.19) |

Notes: 1) SD for BMI and WC were 5.3 and 13.9 overall, 5.8 and 14.5 for women, 5.4 and 13.3 for post-menopausal women and 4.7 and 12.9 for men. 2) Separate models were fitted for each cancer type and adjusted for smoking status, alcohol intake, nationality, the MEDEA deprivation index, type 2 diabetes and had sex and age (5-year categories) in the strata statement. 33) “Main model for BMI + Height” and “Main model for WC + Height” are also adjusted for the height that was used to calculate the BMI of the participants. 4) Brain and CNS include pituitary gland and pineal gland tumors. 5) Models for ovary, cervix and corpus uteri cancers were only computed in women, for breast post-menopausal only in post-menopausal women, and for prostate only computed in men. 6) We only calculated hazard ratios for cancer types for which we ascertained at least 100 cancer cases.

Abbreviations: BMI: Body Mass Index; CI: Confidence Interval; CNS: Central Nervous System; WC: Waist Circumference.

**Table S11.** Distribution of BMI in the SIDIAP database compared to population based-survey data and representative studies.

|  | **Total** | | | | **Men** | | | | | **Women** | | | | |
| --- | --- | --- | --- | --- | --- | --- | --- | --- | --- | --- | --- | --- | --- | --- |
|  | **WHO Categories for BMI** | | | | **Mean** | **WHO Categories for BMI** | | | | **Mean** | **WHO Categories for BMI** | | | |
|  | **Under-** | **Normal** | **Over-** | **Obesity** |  | **Under-** | **Normal** | **Over-** | **Obesity** |  | **Under-** | **Normal** | **Over-** | **Obesity** |
| **Database** | **weight** | **Weight** | **weight** |  |  | **weight** | **Weight** | **weight** |  |  | **weight** | **Weight** | **weight** |  |
| SIDIAP^1^ | 2 | 37.3 | 36.3 | 24.4 | 26.9 | 0.9 | 31.4 | 43.4 | 24.2 | 25.7 | 2.8 | 42 | 30.5 | 24.6 |
| ENPE^2^ | 1.2 | 37.8 | 39.3 | 21.6 | - | 0.5 | 30.2 | 46.5 | 22.8 | - | 2 | 45.4 | 32.1 | 20.5 |
| ENPE Catalonia^2^ | - | - | 39.8 | 15.5 | - | - | - | - | - | - | - | - | - | - |
| Di@bet.es^3^ | - | - | - | 28.2 | - | - | - | - | 28.9 | - | - | - | - | 27.5 |
| ENRICA^4^ | - | - | 39.4 | 22.9 | - | - | - | 46.4 | 24.4 | - | - | - | 32.5 | 21.4 |
| Darios^5^ | - | - | - | - | - | - | 21.3 | 50.7 | 28 | - | - | 36.1 | 35.6 | 28.3 |
| INE Catalonia^6^ | 1.9 | 46.5 | 36.7 | 14.9 | - | 0.4 | 41.8 | 45.5 | 12.3 | - | 3.3 | 51 | 28.3 | 17.5 |
| INE Spain^6^ | 2.2 | 43.3 | 37.1 | 17.4 | - | 0.8 | 36.8 | 44.3 | 18.2 | - | 3.5 | 49.7 | 30 | 16.7 |
| **25-34 years old** |  |  |  |  |  |  |  |  |  |  |  |  |  |  |
| SIDIAP^1^ | 3.2 | 55.4 | 28 | 13.4 | 25.1 | 1.2 | 47.3 | 36.5 | 15 | 23.2 | 4.5 | 60.9 | 22.1 | 12.5 |
| ENPE^2^ | - | - | - | - | 25.8 | 0.3 | 42.3 | 44.4 | 12.7 | 24.5 | 5.2 | 59.2 | 24.3 | 11.4 |
| INE Spain^6^ | - | - | - | - | - | 0.4 | 52.8 | 35.7 | 11.1 | - | 6.9 | 60.8 | 21.8 | 10.6 |
| **35-44 years old** |  |  |  |  |  |  |  |  |  |  |  |  |  |  |
| SIDIAP^1^ | 1.5 | 41.7 | 35.6 | 21.2 | 26.7 | 0.6 | 32.3 | 44 | 23.1 | 24.6 | 2.3 | 50.9 | 27.5 | 19.3 |
| ENPE^2^ | - | - | - | - | 27.1 | 0.8 | 32.3 | 46.8 | 20 | 25.5 | 1.7 | 53 | 26.3 | 19 |
| INE Spain^6^ | - | - | - | - | - | 0.5 | 40 | 43.4 | 16.2 | - | 3.4 | 59.3 | 24.4 | 12.9 |
| **45-54 years old** |  |  |  |  |  |  |  |  |  |  |  |  | 100 |  |
| SIDIAP^1^ | 0.8 | 30.4 | 40.1 | 28.7 | 27.7 | 0.5 | 23.5 | 46.6 | 29.4 | 26.5 | 1.1 | 37 | 34 | 27.9 |
| ENPE^2^ | - | - | - | - | 27.3 | 0.7 | 27.2 | 48.4 | 23.7 | 26 | 1 | 44.8 | 35.4 | 18.8 |
| INE Spain^6^ | - | - | - | - | - | 0.7 | 29.1 | 49.7 | 20.5 | - | 2 | 53 | 29.3 | 15.7 |
| **55-60 years old** |  |  |  |  |  |  |  |  |  |  |  |  |  |  |
| SIDIAP^1^ | 0.5 | 23.1 | 42.9 | 33.5 | 28.1 | 0.4 | 19.3 | 48.2 | 32.1 | 27.8 | 0.6 | 26.7 | 38 | 34.7 |
| ENPE^2^ | - | - | - | - | 28.4 | 0.2 | 20.1 | 44.9 | 35 | 28.1 | 0.5 | 25.7 | 41.6 | 32.1 |
| INE Spain^6^ | - | - | - | - | - | 0.4 | 24.7 | 49.7 | 25.2 | - | 1.3 | 40.5 | 39.5 | 18.8 |
| **18-44 years old** |  |  |  |  |  |  |  |  |  |  |  |  |  |  |
| SIDIAP^1^ | 3.5 | 51.9 | 29 | 15.6 | 25.5 | 1.5 | 43.7 | 37.2 | 17.6 | 23.4 | 5 | 58.2 | 22.8 | 14 |
| ENRICA^4^ | - | - | - | - | 26.5 | - | - | 41.5 | 18.6 | 24.4 | - | - | 13.7 | 11.1 |
| **45-64 years old** |  |  |  |  |  |  |  |  |  |  |  |  |  |  |
| SIDIAP^1^ | 0.6 | 26.4 | 41.7 | 31.3 | 27.9 | 0.4 | 21.2 | 47.7 | 30.7 | 27.3 | 0.8 | 31.2 | 36.2 | 31.8 |
| ENRICA^4^ | - | - | - | - | 28.4 | - | - | 51.9 | 30.9 | 27.3 | - | - | 38 | 24.7 |
| **≥65 years old** |  |  |  |  |  |  |  |  |  |  |  |  |  |  |
| SIDIAP^1^ | 0.6 | 20.7 | 44.4 | 34.3 | 27.8 | 0.4 | 20.3 | 50.9 | 28.4 | 28.5 | 0.7 | 20.9 | 39.9 | 38.5 |
| ENRICA^4^ | - | - | - | - | 28.4 | - | - | 28.9 | 30.6 | 29.1 | - | - | 41.7 | 38.3 |

Notes: 1) SIDIAP (Information System for the Development of Research in Primary Care): Catalonia, data collection period between 2006-2017, n=3.7 million adults, the SIDIAP population is highly representative of the entire Catalan region in terms of geographic, age, and sex distribution, weight and height are recorded as part of routine practice in primary care by general practitioners and nurses. 2) ENPE (Nutritional Study of the Spanish Population): Spain and Catalonia, data collection period between 2014-2015, n=6.800 adults, Cross-sectional study with a representative sample of the non-institutionalized Spanish population, BMI was assessed by trained professionals. 3) Di@bet.es: Spain, data collection between 2009-2010, n=5728, Cross-sectional survey in a representative sample of the Spanish population (participants were selected randomly by conglomerates using the registry of the Spanish health system, BMI was directly assessed. 4) ENRICA (Nutrition and cardiovascular risk study): Spain, data collection between 2008-2009, n=11,991, Cross-sectional study with a representative sample of the non-institutionalized Spanish adult population (sampling was done stratifying provinces and house size), direct BMI measurements by trained nurses. 5) Darios: Spain, data collection between 2000-2009, n=28,743, data from 11 Spanish studies, Direct anthropometric measurements. 6) INE (Spanish Institute of Statistics): Spain and Catalonia, data collection in 2017, n=37000 homes, National survey, representative of the Spanish population, Weight and height are auto reported.

References:

2. Aranceta-Bartrina J, Pérez-Rodrigo C, Alberdi-Aresti G, Ramos-Carrera N, Lázaro-Masedo S. Prevalencia de obesidad general y obesidad abdominal en la población adulta española (25–64 años) 2014–2015: estudio ENPE. Rev Española Cardiol. 2016;69(6):579-587. doi:10.1016/j.recesp.2016.02.010

3. Valdés S, García-Torres F, Maldonado-Araque C, et al. Prevalencia de obesidad, diabetes mellitus y otros factores de riesgo cardiovascular en Andalucía. Comparación con datos de prevalencia nacionales. Estudio Di@bet.es. Rev Española Cardiol. 2014;67(6):442-448. doi:10.1016/j.recesp.2013.09.031

4. Gutiérrez-Fisac JL, Guallar-Castillón P, León-Muñoz LM, Graciani A, Banegas JR, Rodríguez-Artalejo F. Prevalence of general and abdominal obesity in the adult population of Spain, 2008–2010: the ENRICA study. Obes Rev. 2012;13(4):388-392. doi:doi:10.1111/j.1467-789X.2011.00964.x

5. Fernández-Bergés D, Cabrera de León A, Sanz H, et al. Síndrome metabólico en España: prevalencia y riesgo coronario asociado a la definición armonizada y a la propuesta por la OMS. Estudio DARIOS. Rev Española Cardiol. 2012;65(3):241-248. doi:10.1016/j.recesp.2011.10.015

6. Instituto Nacional de Estadística. Sociedad / Salud / Encuesta Nacional de Salud / Determinantes de la salud. Cifras relativas / Características físicas. https://www.ine.es/dynt3/inebase/es/index.htm?type=pcaxis&path=/t15/p419/a2017/p06/&file=pcaxis. Published 2017.

**Table S12.** Association between body mass index and the risk of 26 cancer types: hazard ratios (99% CIs) in the adjusted model, by sex.

|  | **HR (99%CI) per 1 unit increase in BMI** | | | | | |
| --- | --- | --- | --- | --- | --- | --- |
| **Cancer Type** | **Cancer Cases** | **Adjusted (Model 2) Overall** | **Cancer**  **Cases** | **Adjusted (Model 2) Women** | **Cancer**  **Cases** | **Adjusted (Model 2) Men** |
| Head and neck (C00-C14) | 5,199 | 0.94 (0.90-0.98) | 2,008 | 1.04 (0.99-1.10) | 3,191 | 0.85 (0.80-0.90) |
| Esophagus (C15) | 1,560 | 0.88 (0.81-0.95) | 279 | 0.82 (0.70-0.97) | 1,281 | 0.89 (0.82-0.98) |
| Stomach (C16) | 4,897 | 0.97 (0.93-1.01) | 2,120 | 0.99 (0.93-1.04) | 2,777 | 0.95 (0.90-1.01) |
| Colorectal (C18-C21) | 30,886 | 1.06 (1.04-1.08) | 13,086 | 1.03 (1.01-1.06) | 17,800 | 1.10 (1.07-1.13) |
| Liver (C22) | 5,611 | 0.99 (0.95-1.02) | 1,905 | 0.96 (0.91-1.02) | 3,706 | 1.01 (0.96-1.06) |
| Gallbladder & biliary tract (C23-C24) | 1,355 | 1.10 (1.03-1.19) | 724 | 1.13 (1.03-1.24) | 631 | 1.07 (0.94-1.21) |
| Pancreas (C25) | 4,274 | 0.98 (0.94-1.02) | 2,243 | 0.99 (0.94-1.04) | 2,031 | 0.97 (0.91-1.04) |
| Larynx (C32) | 2,974 | 0.83 (0.78-0.88) | 259 | 0.89 (0.76-1.05) | 2,715 | 0.82 (0.77-0.87) |
| Trachea, bronchus & Lung (C33-C34) | 15,455 | 0.83 (0.81-0.85) | 3,186 | 0.87 (0.83-0.91) | 12,269 | 0.82 (0.79-0.84) |
| Bone and articular cartillage (C40-C41) | 766 | 1.00 (0.91-1.10) | 404 | 1.00 (0.88-1.13) | 362 | 1.00 (0.85-1.17) |
| Malignant melanoma of skin (C43) | 6,834 | 0.98 (0.94-1.01) | 3,820 | 0.95 (0.91-0.99) | 3,014 | 1.03 (0.98-1.09) |
| Breast pre-menopause (C50) | 651 | 0.93 (0.84-1.02) | 651 | 0.93 (0.84-1.02) | - | - |
| Breast post-menopause (C50) | 18,995 | 1.07 (1.05-1.08) | 18,995 | 1.07 (1.05-1.08) | - | - |
| Cervix Uteri (C53) | 1,838 | 1.01 (0.95-1.07) | 1,838 | 1.01 (0.95-1.07) | - | - |
| Corpus Uteri (C54-C55) | 5,115 | 1.49 (1.45-1.53) | 5,115 | 1.49 (1.45-1.53) | - | - |
| Ovary (C56) | 2,156 | 0.99 (0.94-1.05) | 2,156 | 0.99 (0.94-1.05) | - | - |
| Prostate (C61) | 26,674 | 0.97 (0.95-0.99) | - | - | 26,674 | 0.97 (0.95-0.99) |
| Testis (C62) | 760 | 0.89 (0.80-1.00) | - | - | 760 | 0.89 (0.80-1.00) |
| Kidney (C64) | 6,124 | 1.16 (1.12-1.20) | 2,313 | 1.15 (1.10-1.21) | 3,811 | 1.17 (1.12-1.23) |
| Bladder (C67) | 20,013 | 0.98 (0.96-1.00) | 3,413 | 0.95 (0.91-1.00) | 16,600 | 0.99 (0.96-1.01) |
| Brain and CNS (C70-C72, C75.1-C75.3) | 3,569 | 1.05 (1.00-1.09) | 1,755 | 1.06 (1.00-1.12) | 1,814 | 1.02 (0.95-1.10) |
| Thyroid (C73) | 2,688 | 1.08 (1.03-1.13) | 2,164 | 1.08 (1.02-1.13) | 524 | 1.11 (0.98-1.25) |
| Hodgkin lymphoma (C81) | 746 | 1.06 (0.97-1.17) | 356 | 1.10 (0.97-1.25) | 390 | 1.01 (0.87-1.17) |
| Non-Hodgkin Lymphoma (C82-C86, C96) | 6,626 | 1.04 (1.01-1.08) | 3,451 | 1.05 (1.01-1.10) | 3,175 | 1.03 (0.98-1.09) |
| Multiple myeloma (C90) | 3,270 | 1.09 (1.04-1.15) | 1,670 | 1.09 (1.03-1.16) | 1,600 | 1.09 (1.01-1.18) |
| Leukemia (C91-C95) | 6,842 | 1.07 (1.04-1.11) | 3,345 | 1.06 (1.01-1.10) | 3,497 | 1.09 (1.03-1.15) |

Notes: 1) The adjusted model (Model 2), which was used in our main analyses, includes BMI as a linear term and is adjusted for smoking status, alcohol intake, nationality, the MEDEA deprivation index, type 2 diabetes and has sex and age (5-year categories) in the strata statement. 2) Brain and CNS include pituitary gland and pineal gland tumors.

Abbreviations: BMI: Body Mass Index; CI: Confidence Interval; CNS: Central Nervous System; GP: General Practitioner; HR: Hazard Ratio.

**Appendix S2.** STROBE Statement-Checklist of items that should be included in reports of cohort studies.

|  | Item No | Recommendation | Page  No |
| --- | --- | --- | --- |
| **Title and abstract** | 1 | (a) Indicate the study’s design with a commonly used term in the title or the abstract | 1, 3 |
|  |  | (b) Provide in the abstract an informative and balanced summary of what was done and what was found | 3 |
| **Introduction** | | | |
| Background/rationale | 2 | Explain the scientific background and rationale for the investigation being reported | 5 |
| Objectives | 3 | State specific objectives, including any prespecified hypotheses | 5 |
| **Methods** | | | |
| Study design | 4 | Present key elements of study design early in the paper | 5 |
| Setting | 5 | Describe the setting, locations, and relevant dates, including periods of recruitment, exposure, follow-up, and data collection | 5 |
| Participants | 6 | (a) Give the eligibility criteria, and the sources and methods of selection of participants. Describe methods of follow-up | 5-6 |
|  |  | (b) For matched studies, give matching criteria and number of exposed and unexposed | - |
| Variables | 7 | Clearly define all outcomes, exposures, predictors, potential confounders, and effect modifiers. Give diagnostic criteria, if applicable | 6-7, Apen. S1, Table S1 |
| Data sources/ measurement | 8* | For each variable of interest, give sources of data and details of methods of assessment (measurement). Describe comparability of assessment methods if there is more than one group | 5-7, Table S1 |
| Bias | 9 | Describe any efforts to address potential sources of bias | 8 |
| Study size | 10 | Explain how the study size was arrived at | - |
| Quantitative variables | 11 | Explain how quantitative variables were handled in the analyses. If applicable, describe which groupings were chosen and why | 5-7 |
| Statistical methods | 12 | (a) Describe all statistical methods, including those used to control for confounding | 7, Fig S1 |
|  |  | (b) Describe any methods used to examine subgroups and interactions | 7 |
|  |  | (c) Explain how missing data were addressed | 7 |
|  |  | (d) If applicable, explain how loss to follow-up was addressed | - |
|  |  | (e) Describe any sensitivity analyses | 8 |
| **Results** | | | |
| Participants | 13* | (a) Report numbers of individuals at each stage of study—eg numbers potentially eligible, examined for eligibility, confirmed eligible, included in the study, completing follow-up, and analysed | 8-9 |
|  |  | (b) Give reasons for non-participation at each stage | 8 |
|  |  | (c) Consider use of a flow diagram | Fig. 1 |
| Descriptive data | 14* | (a) Give characteristics of study participants (eg demographic, clinical, social) and information on exposures and potential confounders | 8-9, Table 1 |
|  |  | (b) Indicate number of participants with missing data for each variable of interest | Table 1 |
|  |  | (c) Summarise follow-up time (eg, average and total amount) | 8-9, Table 1 |
| Outcome data | 15* | Report numbers of outcome events or summary measures over time | 8-9, Fig. 2 |
| Main results | 16 | (a) Give unadjusted estimates and, if applicable, confounder-adjusted estimates and their precision (eg, 95% confidence interval). Make clear which confounders were adjusted for and why they were included | 8-9. Table s2, Fig. 2 |
|  |  | (b) Report category boundaries when continuous variables were categorized | - |
|  |  | (c) If relevant, consider translating estimates of relative risk into absolute risk for a meaningful time period | - |
| Other analyses | 17 | Report other analyses done—eg analyses of subgroups and interactions, and sensitivity analyses | 9-10 |
| **Discussion** | | | |
| Key results | 18 | Summarise key results with reference to study objectives | 11 |
| Limitations | 19 | Discuss limitations of the study, taking into account sources of potential bias or imprecision. Discuss both direction and magnitude of any potential bias | 11-12 |
| Interpretation | 20 | Give a cautious overall interpretation of results considering objectives, limitations, multiplicity of analyses, results from similar studies, and other relevant evidence | 12-13 |
| Generalisability | 21 | Discuss the generalisability (external validity) of the study results | 11 |
| Key results | 18 | Summarise key results with reference to study objectives | 13 |
| **Other Information** | | | |
| Funding | 22 | Give the source of funding and the role of the funders for the present study and, if applicable, for the original study on which the present article is based | 14 |
